# Proximal Effects of a Transdiagnostic, Ecological Momentary Intervention for Enhancing Resilience in Help-Seeking Young People (EMIcompass): Findings From a Secondary Analysis of an Exploratory Randomized Controlled Trial

**DOI:** 10.2196/77150

**Published:** 2026-09-03

**Authors:** Jessica Gugel, Christian Rauschenberg, Isabell Paetzold Jaehme, Anita Schick, Eva Wierzba, Dusan Hirjak, Jan Rasmus Boehnke, Benjamin Boecking, Ulrich Reininghaus

**Affiliations:** 1 Department of Public Mental Health, Central Institute of Mental Health, Medical Faculty Mannheim, Heidelberg University, J5, Mannheim, 68159, Germany, 49 062117031930; 2 German Center for Mental Health (DZPG), partner site Mannheim-Heidelberg-Ulm, Germany; 3 Department of Psychiatry and Psychotherapy, Central Institute of Mental Health, Medizinische Fakultät Mannheim, Mannheim, Germany; 4 Faculty of Health, University of Dundee, Dundee, United Kingdom; 5 Tinnitus Center, Charité - Universitätsmedizin Berlin, Berlin, Germany; 6 ESRC Centre for Society and Mental Health and Social Epidemiology Research Group, King's College London, London, United Kingdom; 7 Health Service and Population Research Department, Centre for Epidemiology and Public Health, Institute of Psychiatry, Psychology & Neuroscience, King's College London, London, United Kingdom

**Keywords:** digital mobile health intervention, just-in-time adaptive intervention, ecological momentary assessment, EMA, ecological momentary intervention, EMI

## Abstract

**Background:**

Ecological momentary interventions (EMIs) offer a promising strategy for targeting putative mechanisms of mental health problems by delivering real-time, tailored intervention components that adapt to person, moment, and context based on data collected using ecological momentary assessment (EMA). However, most research to date focuses on effects on distal outcomes, at the person level, whereas exploration of processes at the microlevel, that is, proximal effects of EMI components on putative momentary mechanisms and outcomes, remains very limited.

**Objective:**

This study aimed to explore the proximal effects of completing EMI components on momentary mental health mechanisms and outcomes (ie, negative affect, stress, and stress reactivity) at the subsequent EMA among youths with early mental health problems.

**Methods:**

EMIcompass is a novel, transdiagnostic, hybrid, compassion-focused EMI for prevention and early intervention of severe mental health conditions in help-seeking youths. Data for our secondary analysis were derived from the experimental condition of the EMIcompass exploratory randomized controlled trial, in which youths aged 14‐25 years with early mental health problems were randomly allocated to the EMIcompass intervention+treatment as usual (experimental condition) or treatment as usual only (control condition). We used self-reported within-subject EMA data collected as part of the EMIcompass intervention in the experimental condition, in which participants were offered EMI tasks and voluntarily decided whether or not to complete them.

**Results:**

Data on proximal effects of EMI interactive task completion on negative affect, stress, and stress reactivity at the subsequent EMA were available in a small subsample of 37 participants. The effect sizes of within-subject proximal effects on momentary negative affect (β=−0.07, 95% CI −0.18 to 0.04; *t*_472.52_=−1.21; *d*-type=−0.12), stress (β=−0.12, 95% CI −0.31 to 0.06; *t*_467.81_=−1.31; *d*-type=−0.14), and stress reactivity (β=−0.04, 95% CI −0.15 to 0.06; *t*_464.70_=−0.78; *d*-type=−0.05) were small to moderate and, at the within-subject level, clinically meaningful, but 95% CIs included 0; hence, these effects were not statistically distinguishable from 0 in this small subsample. Moreover, effect sizes of cumulative training frequency on momentary negative affect, stress, and stress reactivity were very small, and 95% CIs included 0 in the small subsample of 37. Effect sizes for proximal effects for individual task types were small (*d*-type=−0.12 to 0.16), but 95% CIs included, again, 0 in an even smaller subsample of 27 participants.

**Conclusions:**

This secondary analysis implemented a novel approach to advancing our understanding of proximal effects within an EMI framework. While our findings illustrate the feasibility of investigating microlevel processes within an EMI framework, they remain equivocal with regard to proximal effects, due to limited power in this small sample. Further research with larger samples and appropriate study designs, such as microrandomized or N-of-1 designs, is needed to clarify how EMI components impact momentary mental health mechanisms and outcomes.

## Introduction

### Background and Rationale

As technology and digital devices are increasingly integrated into our daily lives, they enable innovative methods for collecting data that can yield significant insights for both research and clinical practice [1]. Ecological momentary assessment (EMA) [2], also known as experience sampling methodology [3], allows us to capture momentary symptoms and mental states as they naturally occur in the flow of individuals’ everyday lives [2,4-7]. EMA can be implemented through dedicated smartphone apps in the form of structured digital diary techniques, allowing users to complete brief questionnaires about their symptoms, mood, and context at specific moments [8]. EMA, thereby, provides intensive longitudinal data on experience and behavior and their fluctuations in the context of everyday life, minimizing the risk of biases, including recall bias [9-11].

Furthermore, EMA principles and techniques can be used to inform digital mental health interventions. Ecological momentary interventions (EMIs) represent an innovative approach, enabling real-time delivery of intervention components tailored to person, moment, and context based on EMA data and the use of interactive or adaptive delivery schemes [12-15]. Interventions that deliver support in daily life and adapt intervention content and delivery to an individual’s momentary state or context are also commonly discussed within the framework of “just-in-time adaptive interventions” [16]. Due to the high availability of digital tools, innovative digital mental health interventions may help improve reach [17]. Given that many mental health conditions have their onset during adolescence [18], digital interventions may be a particularly promising approach for prevention and intervention for youths [19]. However, more research is needed in order to elucidate mechanisms of change to inform the development of targeted innovations with the potential to enhance intervention effects [20]. EMIs can be used to specifically target putative mechanisms that have been linked to the development and persistence of mental health conditions [4]. Possible mechanisms include emotion regulation [21,22] and stress reactivity, referring to an increased negative affect in response to minor stressors in daily life [23-25]. Individuals at risk for various mental health conditions, including depression and psychosis, have been found to have increased stress reactivity [23,24,26-30]. Evidence further suggests that stress reactivity may be an important mechanism involved in linking exposures to socioenvironmental risk factors (eg, childhood adversity) and mental health conditions [24,31].

The “EMIcompass” intervention, a transdiagnostic, compassion-focused, hybrid EMI for improving resilience in youths with early mental health problems, was specifically designed to building on previous findings on EMA and EMI [4,9,14] as well as transdiagnostic momentary mechanisms [13,24,32-34] to target psychological distress, clinical symptoms, and candidate mechanisms [35,36]. It was delivered within a 6-week period in addition to treatment as usual to individuals allocated to the experimental condition [35-38] by trained psychologists under regular supervision. All available health services in Germany, which individuals received, were considered as treatment as usual, including their general practitioner, psychiatrist, and clinical psychologist, except treatment using elements of third-wave cognitive behavioral therapy. A structured manual was developed based on existing manuals, expert consultation, and clinical experience, which guided the selection and sequencing of intervention elements (for further information, see Paetzold et al [37]). The EMIcompass intervention consisted of a 6-week compassion-focused EMI and 4 biweekly sessions (3 training sessions and 1 review session) as well as an optional on-demand session with a duration of 45‐60 minutes. All sessions were delivered to the participants individually, either face-to-face or using a certified and encrypted videoconferencing system. EMIcompass followed a compassion-focused approach [39,40], in which participants were offered breathing exercises, soothing imagery, and self-compassionate imagery and writing techniques in the first 3 training sessions. All sessions offered the opportunity to reflect on the progress and problems participants faced with the EMI. The last session was used to review progress with all tasks and to assess subjective improvement in compliance, satisfaction, and acceptance of the EMIcompass intervention. The smartphone-based EMI was administered through a smartphone-based app (movisensXS, movisens GmbH) on study devices and translated the training from the intervention sessions into individuals’ daily lives [37]. Based on the trained psychologists’ impression in the sessions and the participants’ experiences with the intervention tasks in the first 2 weeks of the intervention, participants were allocated to the basic or the elaborate track of the intervention [37] (for further information, see Further Information on Exploratory Analyses section in Multimedia Appendix 1). After the intervention period, participants were asked to return the study devices and no longer had access to the app.

The EMIcompass EMI offered tasks based on three different delivery schemes (see Paetzold et al [37] for the full manual): (1) Enhancing tasks introduced one new compassion-focused task per week. (2) Consolidating tasks sent user-defined daily reminders to practice tasks that were previously introduced and gave the possibility to complete exercises on-demand. They were added based on meta-analytic evidence indicating that homework compliance contributes to symptom reduction in cognitive behavior therapy studies [41-43]. (3) Interactive tasks were offered to guide participants to use previously learned techniques in moments of high stress or negative affect as detected by voluntary additional EMA. This reflects an important rationale of an EMI: offering tasks in the flow of daily life at a time when the user needs them the most [9,15]. In each delivery scheme, participants were able to decline the EMI tasks.

In an exploratory parallel-group, assessor-blind randomized controlled trial (RCT), first evidence on feasibility of trial methodology and intervention delivery as well as initial signals of efficacy on candidate mechanisms (ie, emotional reactivity, stress reactivity, threat anticipation, resilience, and aberrant salience) and outcomes (ie, quality of life and Brief Psychiatric Rating Scale total scores) of EMIcompass was observed [35]. While previous research shows that compassion-focused interventions may have proximal effects on mental health mechanisms and outcomes in laboratory settings [44,45], to our knowledge, there is currently no evidence of proximal effects on negative affect, stress, and stress reactivity in real-life settings. Examining proximal effects may be crucial to improve existing and develop new EMIs in the future. However, evidence on the feasibility of such a microlevel approach and initial signals of proximal effects of EMI completion on putative momentary mechanisms and outcomes is pending.

### Objectives

Against this background, the aim of this study was to explore proximal effects of the EMIcompass intervention at the microlevel, that is, proximal effects of completing EMI components on momentary mental health mechanisms and outcomes in youths with early mental health problems. We posited that for participants in the experimental condition of the EMIcompass trial, momentary negative affect, stress, and stress reactivity at the subsequent EMA would be lower at observations following the completion of EMI interactive tasks compared to noncompletion (when controlled for momentary negative affect, stress, and stress reactivity at the preceding EMA). The following exploratory hypotheses were tested (focusing on measures of effect size):

- Hypothesis 1: Momentary negative affect will, on average, be lower at the subsequent EMA after EMI interactive task completion when compared to task noncompletion in moments of high stress or negative affect, when controlled for momentary negative affect at the preceding EMA, time between EMAs, clinical staging, age, gender, context change between EMAs, and task time.
- Hypothesis 2: Momentary stress will, on average, be lower at the subsequent EMA after EMI interactive task completion when compared to task noncompletion in moments of high stress or negative affect, when controlled for momentary stress at the preceding EMA, time between EMAs, clinical staging, age, gender, context change between EMAs, and task time.
- Hypothesis 3: Momentary stress reactivity will, on average, be lower at the subsequent EMA after EMI interactive task completion when compared to task noncompletion in moments of high stress or negative affect, when controlled for momentary stress reactivity at the preceding EMA, time between EMAs, clinical staging, age, , context change between EMAs, and task time.

In additional exploratory analyses, we aimed to investigate the potential role of cumulative training effects on the impact of interactive task completion on outcomes, that is, the extent to which the momentary negative affect, stress, and stress reactivity at the subsequent EMA following tasks completion were associated with the total number of tasks completed, while controlling for momentary negative affect, stress, and stress reactivity at the preceding EMA. Moreover, we examined differences in active use by task types by investigating how often the different tasks were selected by our participants. Finally, we investigated whether, for those who completed interactive tasks during moments of high stress or negative affect, outcomes on momentary negative affect, stress, and stress reactivity differed depending on the overarching task type (ie, summarized by content, ie, reflection task, breathing exercises, soothing imagery, or self-compassion tasks).

## Methods

### Patient Public Involvement

In the EMIcompass RCT, youths with early mental health problems aged 14‐25 years were recruited from mental health services at the Central Institute of Mental Health in Mannheim, Germany, as well as local registries, online, and social media advertisements. Based on a modified version of the clinical staging model [46], early mental health problems were classified as current psychological distress (stage 1a), clinical high at-risk mental state (CHARMS; state 1b), or a first treated episode of severe mental disorder (stage 2). To incorporate the participants’ experiences with EMIcompass in the research process and to inform the development of a definite trial, a process evaluation was performed [38].

### Study Design

For our secondary analyses, we used data from the existing EMIcompass RCT, in which participants were randomly allocated to the EMIcompass intervention in addition to treatment as usual (experimental condition) or a control condition of treatment as usual only, including routine mental health care, based on a 50:50 ratio. The EMIcompass RCT was conducted from August 2019 to September 2021 and has been registered in the German Clinical Trials Register (DRKS00017265) [47] on July 31, 2019. The first participant was enrolled on August 7, 2019. Further details on the study protocol [36], main [35], and other findings [37,38,48] are presented elsewhere. The current secondary analysis was registered on the Open Science Framework prior to accessing and analyzing the data [49]. Deviations from the preregistration are shown in the Deviations from the Preregistration section in Multimedia Appendix 1. We used the CONSORT (Consolidated Standards of Reporting Trials) reporting checklist [50] and the CONSORT-EHEALTH (Consolidated Standards of Reporting Trials of Electronic and Mobile Health Applications and Online Telehealth) checklist [51] for reporting our secondary analysis of an exploratory RCT. The CONSORT-EHEALTH checklist is included as Checklist 1.

### Study Setting

Data for the EMIcompass RCT were collected at the Central Institute of Mental Health in Mannheim, Germany. In our secondary analyses, we used data from the intervention period, in which participants used an EMI in their everyday lives.

### Eligibility Criteria

The inclusion criteria were (1) aged 14‐25 years; (2) meeting criteria for stage 1a (Kessler Psychological Distress Scale score of ≥20 [52,53]), stage 1b (CHARMS), or stage 2 (first episode of psychotic, bipolar, severe depressive, or severe anxiety disorder) in accordance with the clinical staging model by Hartmann et al [46]; (3) high stress reactivity assessed with a 2-item self-report measure or the Comprehensive Assessment of At-Risk Mental State subscale on impaired tolerance to everyday stress [54]; (4) reduced positive affect or increased negative affect based on normative scores [55] of the Positive and Negative Affect Scale [56]; (5) willingness to participate; and (6) ability to provide written informed consent (or consent by caregivers or legal guardians for individuals younger than 18 years of age). The exclusion criteria were primary diagnosis of alcohol or substance abuse or dependence (Structured Clinical Interview for DSM-5 [57]), (2) symptoms precipitated by an organic disease, (3) insufficient language proficiency in German, (4) diagnoses of a learning disability, and (5) acute suicidality (Comprehensive Assessment of At-Risk Mental State score > 4 [54]).

### Ethical Considerations

The EMIcompass RCT received ethics approval from the local ethics committee of the Medical Faculty Mannheim, Heidelberg University (2017‐602N-MA). Participants (and for individuals under the age of 18, participants as well as their caregivers or legal guardians) were asked to provide informed consent. Participation in the study was voluntary, and participants were able to withdraw consent at any given time. Data were treated confidentially and analyzed in a pseudonymized form. Data storage and processing were performed in accordance with the General Data Protection Regulation. Participants were covered by liability and commuting accident insurance for the duration of the study. They received compensation for their time (range 85‐175€; 1€=US $1.16 as of August 14, 2026) and travel expenses, depending on how many times they interacted in the EMAs at the outcome assessments (use of the EMAs or EMI during the intervention period was not compensated). Identification of individual participants is not possible from any part of the paper or supplementary material.

### Measures

#### Sociodemographic and Clinical Assessment

Self-reported age and gender (ie, female, male, and nonbinary) were assessed, and clinical staging (ie, current psychological distress, CHARMS, or first treated episode of severe mental disorder) was rated by blinded assessors based on standardized interviews, self-report, and observer ratings at baseline.

#### Self-Reported EMA Measures

EMA data of momentary affect and stress were assessed 6 times per day on 3 consecutive days per week during the intervention period. Momentary negative affect was assessed using 7 established and validated EMA items [24]: “I feel guilty,” “I feel insecure,” “I feel anxious,” “I feel angry,” “I feel down,” “I doubt myself,” and “I am disappointed by myself.” These were rated on a 7-point Likert scale ranging from 1=not at all to 7=very much. Momentary stress was operationalized as minor disturbances and distinctive unpleasant events, activities, and social situations in daily life in accordance with previous EMA studies [24]. Participants were asked to report the pleasantness of the most important event that has happened to them since the last EMA on a bipolar 7-point Likert scale ranging from −3=very unpleasant to +3=very pleasant, the pleasantness of the activity they performed just before the EMA, and the pleasantness of their current social contact on 7-point Likert scales ranging from 1=very unpleasant to 7=very pleasant. Additionally, they were asked to answer the questions “I’m taking care of myself/I’m taking care of somebody” and “I would prefer to be alone/I would prefer to have company” (1=not at all and 7=very much) as follow-up questions to “Who am I with?.” The threshold for triggering interactive tasks was either high momentary negative affect operationalized as a score of 4 or higher on a 7-point Likert scale ranging from 1 to 7 or high momentary stress operationalized as a score <0 on a bipolar scale ranging from −3 to 3. Momentary stress reactivity (ie, the association between stress and negative affect) was computed in a linear mixed model, with the composite momentary stress measure as the independent variable and negative affect as the outcome variable. From this model, the generated fitted value was fitted for quantifying momentary stress reactivity [24,37]. To minimize social desirability bias, all participants were instructed in detail before and during the EMA assessment.

#### Passive EMI Usage Data

During the EMI period, passive usage data, including date and time when a task was delivered, the delivery scheme (ie, enhancing, consolidating, and interactive), and the task type (ie, self-compassionate writing, experiencing emotions as a wave, discovering their own compassionate self, imagining a compassionate companion, counting their breath, breathing with breaks, finding a calming color, writing a compassionate message, imagining a safe and calm place, and emotional compass) were recorded automatically. Based on these data, we computed the variable “task completion” to indicate that an interactive task has been fully completed in the app (ie, a binary variable, coded 1=yes, 0=no). A variable “time” representing the time span between the preceding EMA and the subsequent EMA following the task was computed, with higher values indicating longer periods of time between time points. A variable “task time” was calculated to specify the time participants required for completing the target EMI task, with higher values indicating a longer completion time. As a proxy for new events occurring between time points, we computed a binary variable “context change,” indicating whether participants changed their activity between the preceding and subsequent EMA (0=no context change between the preceding EMA and the subsequent EMA; 1=context changed). To calculate how many tasks were completed at any point in time during the intervention period, we computed the variable “cumulative training frequency” as the sum score of the variable “task completion,” with a higher score indicating a higher cumulative training frequency.

### Harms

Any adverse events were monitored throughout the study period. Overall, no serious adverse events (ie, any serious incidents that result in death, persistent or significant disability or incapacity that require hospitalization, or life-threatening situations), and only few adverse device effects (eg, problems with the study smartphone or the app), were observed during the trial period. A more detailed overview can be found elsewhere [35].

### Sample Size

For the primary analysis of the EMIcompass RCT, the required sample size was determined with a power simulation [36]. No sample size calculation for our secondary analysis was performed.

### Randomization and Blinding

The current secondary analysis used within-subject data generated in the experimental condition of the EMIcompass RCT. Participants in the EMIcompass RCT were randomized at the person-level at a 50:50 ratio to the experimental or control condition. An independent researcher performed block randomization in blocks of 4 through a computer-generated sequence, with stratification for the 3 stages (ie, stages 1a, 1b, and 2). For the current secondary analysis, no further randomization was performed. The criterion whether or not participants completed an EMI task was not randomized, but participants decided this voluntarily for each initiated interactive task.

### Statistical Methods

Given the exploratory nature of all analyses, 95% CIs and Cohen *d*-type effect sizes (hereafter, *d*-type) were the primary focus for all analyses. Stata (version 17.0; StataCorp) was used for statistical analysis. Due to the hierarchical structure of experience sampling data, clustering of EMA data was controlled for by using linear mixed modeling [58,59]. Mean scores for the momentary outcome variables were centered around the person. For better comparability between findings on different momentary mechanisms or outcomes, between-person *z*-standardization was used for all variables. As different tasks vary in the amount of time required to complete them, task time was *z*-standardized for each task separately. Linear mixed models were fitted using restricted maximum likelihood, allowing for the use of all available data under the relatively unrestrictive assumption that data are missing at random if all variables associated with missing values are included in the model. For all analyses, Kenward-Roger adjustments were applied to calculate more conservative margins of error [60]. To explore whether completion of EMI interactive tasks in moments of high stress or negative affect was associated with, on average, lower negative affect, stress, and stress reactivity at the subsequent EMA when compared to EMI noncompletion, we fitted linear mixed models with the difference variable of each momentary outcome as the outcome variable. Task completion (2-level factor) was included as an independent variable in each model along with the following potential confounding variables: momentary mechanisms and outcomes (ie, negative affect, stress, or stress reactivity, respectively), at the preceding EMA, time between EMAs, clinical staging (3-level factor with clinical stage 1a as the reference category), age, gender (2-level factor, 0=female, 1=male), context change (2-level factor), and task time. Within-subject clustering of repeated measures was taken into account by adding a level-2 random intercept and by allowing the models’ level-1 residuals to be correlated with a completely unstructured error variance-covariance matrix. In all analyses, all data from all participants were used in the analysis, including those who have low adherence to or who dropped out from the intervention. Further, we conducted exploratory analyses of same-day posttask associations using a broader inclusion criterion, including all observations for which at least 1 EMA was completed on the same day after task completion (see Exploratory Analyses of Same-Day Post-Task Associations section in Multimedia Appendix 1). Cohen *d*-type effect sizes were constructed for all regression analyses by dividing the β coefficients by the square root of the total variance in the full models. The magnitude of effect sizes was interpreted in line with previous research, indicating that within-subject effects are unlikely to exceed effect sizes of Cohen *d*=0.2 [61,62].

To examine the extent to which the total number of tasks completed was associated with momentary negative affect, stress, or stress reactivity at the subsequent EMA after interactive task completion, we fitted linear mixed models. Therefore, momentary negative affect, momentary stress, or momentary stress reactivity at the subsequent EMA were each entered as dependent variables in separate models with each cumulative training frequency as an independent variable while controlling for momentary negative affect, stress, or stress reactivity, respectively, at the preceding EMA, time between EMAs, clinical staging, age, gender, context change between EMAs, and task time. To examine differences in active use by task type among participants, we performed descriptive analyses by calculating distributions of relative frequencies by task type. For each participant, we calculated the relative frequencies with which they selected the available tasks and then computed mean scores of those frequencies across all participants. We further examined whether, for those who completed interactive tasks in moments of high stress or negative affect, proximal effects on momentary negative affect, momentary stress, and momentary stress reactivity differed depending on the task type of interactive tasks. Therefore, overarching task types were summarized by content (ie, reflection task and emotional compass), breathing exercises (counting their breath and breathing with breaks), soothing imagery (finding a calming color, imagining a safe and calm place, and experiencing emotions as a wave), and self-compassion tasks (self-compassionate writing, discovering their own compassionate self, imagining a compassionate companion, and writing a compassionate message). We fitted linear mixed models with momentary negative affect, momentary stress, and momentary stress reactivity at the subsequent EMA, each entered in separate models as dependent variables and task type (effect-coded and dummy-coded) as independent variables while controlling for momentary outcomes at the preceding EMA, time between EMAs, clinical staging, age, gender, context change between EMAs, and task time. See Further Information on Exploratory Analyses section in Multimedia Appendix 1 for more statistical details on exploratory analyses.

## Results

### Participant Flow

**Figure 1.**
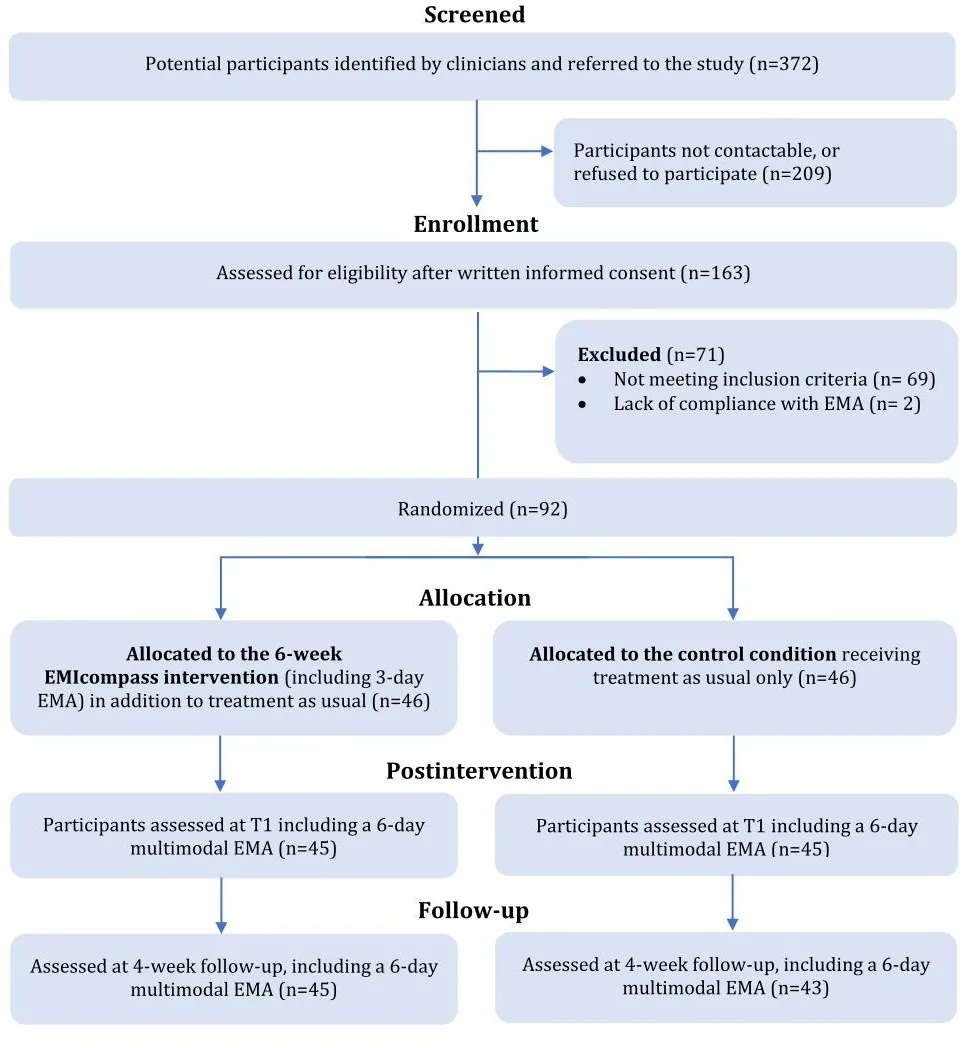
Flow diagram of participants in the EMIcompass RCT according to CONSORT guidelines [50]. For the current secondary analysis, only data from participants allocated to the EMIcompass intervention were included. From the 46 individuals in the EMIcompass experimental condition, 37 participants were included in the current secondary analysis, 5 participants declined the voluntary EMA during the intervention period, and 4 participants could not be included in our secondary analysis as the number of consecutive EMAs completed during the EMI was not sufficient for our analyses (1 of those 4 participants dropped out of the RCT during the intervention period). CONSORT: Consolidated Standards of Reporting Trials; EMA: ecological momentary assessment; EMI: ecological momentary intervention; RCT: randomized controlled trial.

Figure 1 depicts the participant flow of the EMIcompass RCT according to the CONSORT guidelines [50]. For the EMIcompass RCT, 372 individuals were identified for potential participation. Of those, 163 were screened for eligibility. The full sample of 92 participants was randomized and allocated to either the experimental or control condition at a 50:50 ratio. For the current secondary analysis, only participants from the experimental condition were eligible. From the 46 individuals in the EMIcompass experimental condition, 5 participants declined the voluntary EMA during the intervention period, and 4 participants could not be included in our secondary analysis as the number of consecutive EMAs completed during the EMI was not sufficient for our analyses (1 of those 4 participants dropped out of the RCT during the intervention period). Overall, 37 participants were included in our analyses.

### Sample Characteristics

An overview of basic sample characteristics is displayed in Table 1. More details on the full sample of the EMIcompass RCT can be found elsewhere [35,37]. The mean age was 21.38 (SD 2.97; range 14‐25) years, and 81% (30/37) of participants identified as female and 19% (7/37) identified as male. The majority of participants was classified as stage 1a (psychological distress; 21/37, 57%), 27% (10/37) were classified as stage 1b (CHARMS), and 16% (6/37) fulfilled criteria of a first episode of a severe mental health condition (stage 2).

**Table 1.** Basic sample characteristics of the full analytical sample^a^.

Characteristic	Participants (n=37)
Age (years), mean (SD)	21.38 (2.97)
Gender, n (%)	
Female	30 (81)
Male	7 (19)
Other	0 (0)
Clinical staging, n (%)	
Stage 1a	21 (57)
Stage 1b	10 (27)
Stage 2	6 (16)

a Sample sizes varied over the different analyses due to missing ecological momentary assessments.

### Proximal Effect of Task Completion on Momentary Negative Affect (Hypothesis 1)

Table 2 shows the results from the linear mixed model with random intercept for task completion and momentary negative affect. Proximal effects of EMI task completion on momentary negative affect at the subsequent EMA were in the small effect size range in the hypothesized direction, but all 95% CIs included 0 (β=−0.07, 95% CI −0.18 to 0.04; *t*_472.52_=−1.21; *d*-type=−0.12). The 95% CI for the effect of negative affect at the preceding EMA excluded 0, and the effect size indicated a large effect (β=0.63, 95% CI 0.56-0.70; *t*_342.30_=17.81; *d*-type=1.10). Unadjusted effects of task completion on momentary negative affect at the subsequent EMA are reported in Table S4 in Multimedia Appendix 1.

**Table 2.** Proximal effect of task completion on momentary negative affect at the subsequent ecological momentary assessment (EMA) in a linear mixed model^a^^,b,c^.

	β (95% CI)	*t* test (*df*)	*d*-type (95% CI)
Task completion	−0.07 (−0.18 to 0.04)	−1.21 (472.52)	−0.12 (−0.32 to 0.07)
Negative affect at preceding EMA	0.63 (0.56 to 0.70)	17.81 (342.30)	1.10 (0.92 to 1.28)
Time between EMAs	−0.00 (−0.06 to 0.05)	−0.16 (322.38)	−0.01 (−0.11 to 0.09)
Clinical staging: stage 1b	0.09 (−0.21 to 0.39)	0.62 (31.70)	0.16 (−0.34 to 0.66)
Clinical staging: stage 2	0.51 (−0.10 to 0.93)	2.54 (30.30)	0.90 (0.20 to 1.60)
Age	−0.00 (−0.05 to 0.04)	−0.25 (26.09)	−0.01 (−0.08 to 0.06)
Gender	−0.14 (−0.50 to 0.21)	−0.84 (25.16)	−0.25 (−0.84 to 0.34)
Context change between EMAs	0.00 (−0.10 to 0.10)	0.01 (463.45)	0.00 (−0.18 to 0.18)
Task time	−0.03 (−0.10 to 0.05)	−0.767 (471.54)	−0.04 (−0.17 to 0.08)

a Adjusted for negative affect at the preceding EMA, time between EMAs, clinical staging, age, gender, context change between EMAs, and task time.

b A total of 493 observations clustered in 36 participants.

c A multilevel model with random intercept and slope was tested against this model and rejected.

### Proximal Effect of Task Completion on Momentary Stress (Hypothesis 2)

Table 3 presents results from the linear mixed model with random intercept for task completion and momentary stress. The results indicated a small effect size for the proximal effect of EMI task completion on momentary stress at the subsequent EMA in the hypothesized direction, but all 95% CIs included 0 (β=−0.12, 95% CI −0.31 to 0.06; *t*_467.81_=−1.31; *d*-type=−0.14). The 95% CI for the effect of stress at the preceding EMA excluded 0, and the effect size indicated a small effect (β=0.13, 95% CI 0.04-0.23; *t*_476.87_=2.84; *d*-type=0.15). For context change between EMAs, a small negative effect size was observed, but the 95% CI included 0 (β=−0.13, 95% CI −0.30 to 0.04; *t*_481.78_=−1.51; *d*-type=−0.15). Unadjusted effects of task-completion on momentary stress at the subsequent EMA are shown in Table S4 in Multimedia Appendix 1.

**Table 3.** Proximal effect of task completion on momentary stress at the subsequent ecological momentary assessment (EMA) in a linear mixed model^a,b,c^.

	β (95% CI)	*t* test (*df*)	*d*-type (95% CI)
Task completion	−0.12 (−0.31 to 0.06)	−1.31 (467.81)	−0.14 (−0.34 to 0.07)
Stress at preceding EMA	0.13 (0.04 to 0.23)	2.84 (476.87)	0.15 (0.05 to 0.26)
Time between EMAs	0.05 (−0.05 to 0.14)	0.92 (481.94)	0.05 (−0.06 to 0.16)
Clinical staging: stage 1b	0.24 (−0.15 to 0.64)	1.26 (29.13)	0.27 (−0.15 to 0.70)
Clinical staging: stage 2	−0.15 (−0.69 to 0.38)	−0.59 (29.32)	−017 (−0.75 to 0.40)
Age	0.02 (−0.04 to 0.07)	0.65 (23.36)	0.02 (−0.04 to 0.08)
Gender	0.28 (−0.18 to 0.74)	1.26 (23.96)	0.32 (−0.18 to 0.81)
Context change between EMAs	−0.13 (−0.30 to 0.04)	−1.51 (481.78)	−0.15 (−0.33 to 0.04)
Task time	−0.01 (−0.13 to 0.12)	−0.11 (479.36)	−0.01 (−0.15 to 0.13)

a Adjusted for stress at the preceding EMA, time between EMAs, clinical staging, age, gender, context change between EMAs, and task time.

b A total of 492 observations clustered in 36 participants.

c A multilevel model with random intercept and slope was tested against this model and rejected.

### Proximal Effect of Task Completion on Momentary Stress Reactivity (Hypothesis 3)

Table 4 shows findings from the linear mixed model with random intercepts for task completion and momentary stress reactivity at the subsequent EMA. We observed a very small effect size for the proximal effect of EMI task completion on momentary stress reactivity at the subsequent EMA, with the 95% CI including 0 (β=−0.04, 95% CI −0.15 to 0.06; *t*_464.70_=−0.78; *d*-type=−0.05). Unadjusted effects of task completion on momentary stress reactivity at the subsequent EMA are shown in Table S4 in Multimedia Appendix 1.

**Table 4.** Proximal effect of task completion on reductions in momentary stress reactivity in a linear mixed model^a,b,c^.

	β (95% CI)	*t* test (*df*)	*d*-type (95% CI)
Task completion	−0.04 (−0.15 to 0.06)	−0.78 (464.70)	−0.05 (−0.18 to 0.08)
Stress reactivity at the preceding EMA^d^	0.16 (0.08 to 0.25)	3.67 (478.25)	0.19 (0.08 to 0.31)
Time between EMAs	0.02 (−0.04 to 0.08)	0.71 (461.15)	0.02 (−0.04 to 0.09)
Clinical staging: stage 1b	0.47 (−0.13 to 1.07)	1.60 (31.70)	0.56 (−0.13 to 1.25)
Clinical staging: stage 2	1.01 (0.16 to 1.85)	2.43 (30.44)	1.21 (0.21 to 2.20)
Age	−0.03 (−0.12 to 0.05)	−0.84 (30.70)	−0.04 (−0.14 to 0.06)
Gender	−0.55 (−1.31 to 0.21)	−1.48 (28.82)	−0.65 (−1.52 to 0.22)
Context change between EMAs	−0.03 (−0.13 to 0.07)	−0.62 (459.72)	−0.04 (−0.15 to 0.08)
Task time	0.00 (−0.07 to 0.07)	0.04 (455.12)	0.00 (−0.08 to 0.08)

a Adjusted for stress reactivity at the preceding EMA, time between EMAs, clinical staging, age, gender, context change between EMAs, and task time.

b N=492 observations clustered in 36 participants.

c A multilevel model with random intercepts and slopes was tested against this model and rejected.

d EMA: ecological momentary assessment.

### Exploratory Analyses of Same-Day Posttask Associations

In our exploratory analyses of same-day posttask associations using a broader inclusion criterion (ie, including all observations for which at least 1 EMA was completed on the same day after task completion), we observed very small to small effect sizes for the proximal effects of EMI task completion on momentary negative affect (β=−0.06, 95% CI −0.18 to 0.04; *t*_592.77_=−1.21; *d*-type=−0.10; n=620 observations clustered in 37 participants), stress (β=−0.05, 95% CI −0.22 to 0.12; *t*_592.67_=−0.56; *d*-type=−0.05; n=617 observations clustered in 36 participants), and stress reactivity (β=−0.01, 95% CI −0.10 to 0.08; *t*_588.79_=−0.19; *d*-type=−0.01; n=617 observations clustered in 36 participants) at the subsequent EMAs, with all 95% CIs including 0 (see Exploratory Analyses of Same-Day Post-Task Associations section in Multimedia Appendix 1).

### Proximal Effects of Cumulative Training Frequency

Effect sizes of cumulative training frequency on momentary negative affect (β=0.08, 95% CI −0.03 to 0.19; *t*_363.32_=1.42; *d*-type=0.14), momentary stress (β=0.03; *t*_237.34_=0.30, 95% CI −0.14 to 0.20; *d*-type=0.03), and momentary stress reactivity (β=−0.03, 95% CI −0.14 to 0.07; *t*_472.25_=−0.65; *d*-type=−0.04) at the subsequent EMA were very small, and 95% CIs included 0 (for tables, see Tables S5-S7 in Multimedia Appendix 1).

### Differences in Active Use by Task Types

To indicate differences in active use by task types, Table 5 shows mean values across all participants of the relative frequencies in which they actively selected the different tasks that were available to them, based on the study track (general tasks, basic study track, and advanced study track).

**Table 5.** Mean and SD of relative frequencies of completed tasks by task type—general tasks.

	Mean (SD)	Values, n^a^
General study track		
Compass of emotions	0.13 (0.13)	37
Counting your breath	0.57 (0.18)	37
My soothing color	0.09 (0.11)	37
Basic study track		
My calm, safe, and peaceful place	0.16 (0.20)	14
Breathing with breaks	0.21 (0.20)	14
Emotion as a wave	0.45 (0.36)	14
Elaborate study track		
Compassionate image	0.12 (0.12)	22^b^
My calm, safe, and peaceful place	0.20 (0.23)	22^b^
Compassionate self	0.22 (0.25)	22^b^
Compassionate message	0.56 (0.31)	22^b^

a The full analytical sample of 37 participants had access to the tasks in the general study track. From those, a subsample of 14 participants was allocated to the basic study track, and a subsample of 23 participants was allocated to the elaborate study track.

b Missing cases: n=1.

### Differences in Proximal Effect on Momentary Mental Health Outcomes by Overarching Task Types

Effect sizes for proximal effects for individual task types were small (*d*-type=−0.12 to −0.16), but all 95% CIs included 0 (Tables S9-S11 in Multimedia Appendix 1). For the proximal effect on momentary negative affect by overarching task types, the largest effect size was found for breathing exercises (β=−0.12, 95% CI −0.33 to 0.08; *t*_194.05_=−1.17; *d*=−0.19).

## Discussion

### Principal Findings

In our secondary analysis in this small subsample, we found small effect sizes for proximal effects of EMI interactive task completion (ie, tasks delivered in times of high stress or negative affect) on momentary negative affect, stress, and stress reactivity at the subsequent EMA in the hypothesized direction, but the CIs for all effects were broad and included 0 and should therefore be interpreted very cautiously. Further, effect sizes of cumulative training frequency on momentary negative affect, stress, and stress reactivity were very small, and the CIs included 0. Finally, effect sizes for proximal effects for individual overarching task types were small, and CIs included, again, 0. For the proximal effect on momentary negative affect by overarching task types, the largest effect size was found for breathing exercises.

### Interpretation

To our knowledge, this is the first study that reports proximal effects of compassion-focused therapy (CFT)–based EMI components specifically designed to target putative mechanisms in daily life on momentary negative affect, stress, and stress reactivity. Even though there is great variability in content, design, and target populations of EMIs, the current state of research indicates their overall potential in improving mental health outcomes [9,63-67], including findings from the EMIcompass RCT [35]. In addition, research on adaptive EMIs [67] more recently coined just-in-time adaptive interventions and provided insight into the possibility to apply real-time data analyses to predict proximal outcomes [68]. This opens the opportunity to dynamically personalize content and improve adaptive interventions by delivering the right tasks at the right time [9,67,69]. Given that complex microdynamics occurring in daily life may underlie the development of mental health conditions, understanding time-lagged dynamic processes in everyday life and the most suitable interventions to target them are very relevant for improving mental health outcomes [69-72].

In a laboratory setting, first signals of proximal effects of compassion-focused intervention tasks on reduced negative affect following experimentally induced stress were found [44,45]. Moreover, decreased stress reactivity was reported in the EMIcompass pilot study [13] and EMIcompass RCT [35], and reductions in momentary negative affect were reported in its qualitative analyses [38]. Even though the CIs in our analyses included 0, the direction of effects and effect sizes in our real-life data tentatively suggest a putative beneficial effect of task completion on momentary negative affect, such that momentary negative affect at the subsequent EMA may on average be lower at times participants chose to complete tasks versus at times they chose not to complete them. While our current analyses allow for no such conclusion, they underscore the need for future research. Especially in EMA data, even effects in the small effect size range may indicate clinically meaningful change in mental health mechanisms and outcomes, as effects may accumulate over time [73]. Given that Kraiss et al [70] report evidence on substantial carry-over effects of negative affect to the subsequent time point, our findings of associations between momentary outcomes at the preceding EMA and the respective momentary outcomes at the subsequent EMA support further optimization and evaluation of interventions targeting negative affect in daily life.

CFT has shown to reduce stress as a distal outcome in real-life training sessions [74,75] and digital interventions [35,76], with first evidence also suggesting that it may reduce stress reactivity [35,77]. While the CIs in our analyses included 0, patterns in our data suggested task completion and context change as potentially relevant factors for lower momentary stress at the subsequent EMA. This may suggest not only the intervention component as such, but also more stressor-related factors might play a role in stress reduction. Given that stress arises from cognitive appraisal processes in response to a stressor, coping strategies can either address the stressor directly or aim to regulate emotional responses [78]. While CFT-based training focuses on the emotional response [79], future interventions might also initiate participants to address the stressor directly, depending on their current needs.

The EMIcompass interactive tasks aimed to deliver support when individuals need it most in their daily lives, identified based on EMA data [36]. However, compliance with EMA may be influenced by the specific context [80]. Previous research indicates that high stress and negative affect may predict nonresponse to EMA, even though these are precisely the moments when interventions may be most needed [81]. This poses a challenge to delivering and evaluating EMIs, as critical moments may be missed and lead to a biased estimation of potential effects. However, in the EMIcompass hybrid intervention, important criteria of feasibility such as session attendance and EMI task completion per week were met [35].

In further analyses, we aimed to explore when exactly effects might occur, which tasks are most effective and well-received, and whether there are any cumulative training effects. However, we found no beneficial effect of cumulative training frequency on momentary outcomes after task completion. While this is consistent with prior findings from the EMIcompass RCT suggesting no association between cumulative training frequency and psychological distress at postintervention [48], this convergence cannot be interpreted as evidence for the absence of an effect. This may, however, imply that the relationship between training frequency and effects on mental health may be more nuanced than a linear dose-response relationship, aligning with previous research [82]. In addition, frequent reminders for completing EMI components may also be viewed as minor stressors [83,84], highlighting the need to consider individual preferences on the optimal EMI delivery scheme. Moreover, potential habituation, fatigue, or burden effects should be considered when interpreting the role of cumulative training frequency. This may partly be reflected in our data in the positive direction of the proximal effect of cumulative training frequency on momentary negative affect [85]. Given that these estimates were small and imprecise, they should not be interpreted as robust evidence for habituation or fatigue. However, they suggest that future adequately powered studies should examine whether repeated EMI task completion is associated with adaptation, reduced responsiveness, fatigue, or perceived burden over time.

We included age, gender, and clinical staging into the analyses to explore potential differences in the effects on proximal mental health mechanisms and outcomes by sociodemographic characteristics or symptom severity. Due to the unbalanced sample sizes across the clinical stages, the interpretation of any effects of clinical staging on our results is limited. However, possible differences by symptom severity may be considered in future research. Previous research suggests that participants with more severe symptoms may experience more negative affect and stress [86], which may partly be reflected in our data, as stage 2 was associated with higher momentary stress at the subsequent EMA. However, symptom severity may also be associated with greater improvements in various mental health outcomes [87].

We further sought to investigate which tasks participants tend to use more frequently and whether those were the ones yielding the strongest effects. While previously reported findings from the EMIcompass feasibility RCT [35] and findings on subjective ratings of participants’ satisfaction with the different tasks assessed at postintervention reported in this study (Table S12 in Multimedia Appendix 1) indicated high general satisfaction with all EMI components, breathing, emotion regulation, and compassion-focused EMI components were selected most frequently. Given that engagement in digital interventions and real-world uptake shows high variability [88-90], it is crucial to further examine how to develop appealing tasks individuals are prepared to use in their daily life. Moreover, effect sizes for proximal effects for individual task types on momentary outcomes in moments of high stress or negative affect were very small and not statistically distinguishable from 0. For the proximal effect on momentary negative affect, the largest point estimate was found for breathing exercises. Although this should be interpreted with caution, previous research highlights that breathing exercises can decrease tension or discomfort [91-93]. Given our limited sample size, any potential differences between tasks should be interpreted with caution but rather explored further in future research.

As a CFT-based EMI, EMIcompass aimed to help individuals understand and manage the threat, drive, and soothing system [35]. Breathing exercises may physiologically activate the parasympathetic nervous system, and thus be an effective way to activate the soothing system even in high-stress situations, potentially yielding short-term effects [94,95]. Different EMI tasks may initiate various mechanisms of change, which may be reflected in differing temporal dynamics or other proximal outcome variables that were not included in our analyses [86]. Thus, plus given the limited statistical power, null findings cannot be interpreted as the absence of microlevel effects. Building on this, microlevel processes in interventions are important, and trends within them should be considered to understand how long-term effects of interventions arise. Moreover, needs and preferences vary across individuals and situations [96-98], highlighting the need for flexibility within interventions rather than a “one-size-fits-all” approach [69].

It is important to note that the reduction of symptoms alone may not sufficiently reflect the mechanisms addressed by a CFT-based intervention [39,40,99]. The primary aim of CFT is not to reduce negative affect but rather to encourage acceptance while enhancing a compassionate mindset that helps individuals cope with it [39,99]. Such a perspective on therapeutic processes can also be found in other third-wave psychotherapy approaches such as acceptance and commitment therapy [100,101]. Previous qualitative findings underpin this idea, as participants particularly mentioned changes in their perspective on emotional states or increased self-compassion beyond the mere reduction of momentary negative affect [38,84]. While our analyses with the available data may help our understanding of microlevel effects and processes of CFT-based EMIs such as EMIcompass, this approach is not optimal for evaluating the effectiveness of such interventions. The selection of proximal outcomes was guided by the EMIcompass intervention logic model and the published trial protocol [36]. In the protocol, stress reactivity was defined as the primary candidate mechanism, and momentary stress and negative affect were specified as central EMA domains. In addition, interactive EMI tasks were triggered by elevated momentary stress or negative affect. Thus, examining subsequent momentary negative affect, stress, and stress reactivity was conceptually aligned with the intended proximal targets of the intervention. Nevertheless, we acknowledge that these outcomes do not fully capture all CFT-specific proximal mechanisms, such as compassionate responding, self-compassion, acceptance, safeness, soothing, or distraction. Future studies should therefore include these constructs as momentary proximal outcomes to more directly test the mechanisms through which compassion-focused EMI components may exert their effects in daily life. In addition, a shift in one’s attitude toward emotions and a more compassionate approach to oneself may alter individuals’ ways of engaging with new situations, whether or not a task has been performed before [102]. This may be accompanied by changes in the subjective evaluation of situations, that is, over time, the same situation would induce less stress or negative affect [103].

### Limitations

An important strength of this study is the focus on proximal effects of EMI components on momentary mental health mechanisms and outcomes in real life using EMA and EMI data. However, several methodological considerations and limitations should be taken into account when interpreting findings. First, proximal effects in this study referred to the EMA following an EMI interactive task, resulting in varying time lags, as EMAs were scheduled at random within set blocks of time [36]. Hence, important effects directly after task completion may have been missed. However, all analyses were controlled for time between EMAs.

Second, the EMIcompass feasibility trial was not powered for this secondary analysis. Power requirements in EMA studies depend on several design parameters, including the number of participants, the number of observations per participant, the intraclass correlation, missingness, the expected effect size, and the complexity of the statistical model. Prior simulation work suggests that, depending on the study design and expected effect sizes, detecting small within-person effects may require at least 60 individuals with a minimum of 30 EMA observations each [104]. Future trials should estimate design-specific target sample sizes using a priori power analyses, for example, with tools such as the Shiny app [105]. Due to limited statistical power, our findings should be interpreted with caution. In particular, nonsignificant findings should not be taken as evidence for the absence of an effect, while observed effect-size estimates should also not be overinterpreted, as estimates from underpowered studies may be highly unstable [106,107]. Thus, the observed effects should be regarded as descriptive and hypothesis-generating rather than as precise estimates of the true effect. Effect sizes are reported to facilitate interpretation and comparison with prior work, but they should be interpreted cautiously and in light of the limited precision of the present study. Where applicable, we considered effect sizes in relation to previous research, suggesting that traditional benchmarks for Cohen *d* might not be directly applicable to within-subject effects [61,62]. At the same time, it is important to note that effect size estimates in underpowered studies may be highly unstable. In addition to the EMIcompass feasibility trial not being powered for the current analyses, only participants from the EMIcompass experimental condition were included in our analyses, and 9 participants were not eligible to be included in the current analyses. Moreover, we used a rather conservative exclusion criterion for our main analyses by excluding all observations for which the EMA following the interactive task was not completed, further limiting the sample size and power. We conducted exploratory analyses of same-day posttask associations using a broader inclusion criterion, including all observations for which at least 1 EMA was completed on the same day after task completion (Tables S1-S3 in Multimedia Appendix 1). These analyses included a greater number of EMA observations, while the number of participants remained unchanged; however, statistical power remained limited. Importantly, these analyses differ from the primary analysis because they no longer capture effects at the immediate subsequent EMA, but instead reflect broad same-day posttask associations. Accordingly, they should not be interpreted as direct robustness checks of the primary analyses. Future research with adequate statistical power is needed to examine both immediate proximal effects and their temporal dynamics over longer posttask windows.

Third, it is important to critically evaluate the sample selection. The EMIcompass sample predominantly consisted of young female individuals, limiting generalizability of findings to young male and nonbinary individuals. This difference might be partly explained by differences in the prevalence of symptoms or diagnoses, with depression and anxiety being more prevalent in female individuals [108], and primary substance use disorders, that were excluded in the EMIcompass trial, being more prevalent in male individuals [109]. Furthermore, our sample included participants from different developmental stages. While all analyses were controlled for age, conclusions regarding age effects should be drawn cautiously and explored in adequately powered future studies.

Fourth, participants were either allocated to a basic or elaborate study track in the EMIcompass intervention, which may have distorted results in our analyses about task type differences. Differences across various levels of intervention complexity should be examined in future studies to enable the development of optimally effective intervention tasks for different individuals.

Fifth, multiple potential sources of confounding in real-life data need to be acknowledged. We controlled for time between EMAs, clinical staging, age, gender, context change between EMAs, and task time. However, these variables may not fully capture the real-life circumstances that influence whether participants chose to complete an EMI task, particularly during high-stress moments; for example, context change may not fully account for the various events that may occur between EMAs in daily life. Thus, task completion time points may differ systematically from noncompletion time points in ways that were not measured by or included in our covariates. This potential self-selection and unmeasured time-varying confounding limits interpretation of the observed associations. Moreover, task completion might underestimate the time spent with EMI components, since we lack data on tasks completed independently without using the app, as observed in a previous study [110]. This should be considered in future studies by including a short question at the beginning of each EMA, asking whether or not participants have completed any task by themselves, without using the smartphone. Assessing training without the smartphone would also allow for a more accurate assessment of training effects. In addition, including large numbers of putative confounders into analyses may limit statistical power [111], which should be considered in future research. However, we calculated crude estimates that indicated that the overall patterns of our findings remained consistent (Table S4 in Multimedia Appendix 1).

Sixth, the high number of analyses performed may have increased the probability of identifying spurious patterns. Although the primary focus of the current exploratory analyses was on effect size, this limitation needs to be taken into account and warrants caution when interpreting our findings.

Seventh, given that the current analyses rely on self-reported data, a risk for social desirability bias may exist. While we sought to minimize this risk by using standardized EMA instructions, this bias cannot be ruled out and should be considered when interpreting the findings. Moreover, our operationalization of stress using an established EMA measure may not fully capture all facets of stress. Future research may complement self-report measures with more objective indicators such as heart-rate variability [112].

Eighth, we clustered task types for our analyses on differences in proximal outcomes by task types. The clustering was based on theoretical assumptions regarding underlying therapeutic mechanisms of change, but these assumptions were not empirically tested within our study. Hence, heterogeneity within clusters may remain.

Finally, the EMIcompass trial was conducted, and EMA data collected during the COVID-19 pandemic, which might have altered participants’ daily lives through changes like restricted social contacts, heightened health-related stress, and a shift from university or work to home-office settings [113,114]. Consequently, our data may, in some cases, depict experiences and contexts in times of crises rather than in typical everyday life.

### Conclusions

This secondary analysis of the EMIcompass feasibility RCT implemented an innovative approach to examine proximal effects of completion of compassion-focused EMI components on momentary mechanisms and outcomes. We found no proximal effects in our small sample, but patterns in our data tentatively suggest that EMI task completion may be associated with lower negative affect, stress, and stress reactivity at the subsequent EMA, warranting further investigation. While our findings remain equivocal due to limited power in this small sample, our secondary analysis did demonstrate the feasibility of investigating microlevel processes within an EMI framework and move beyond a purely descriptive account of proximal change. Furthermore, this study illustrated approaches to examine individual task types and the effect of training frequency on proximal effects. However, the current analysis does not allow for conclusions to be drawn about the relationship between proximal and distal outcomes. Further evidence on within-person shifts in EMI studies and their potential relevance for longer-term outcomes is needed. Rather than relying solely on between-group comparisons to detect whether or not an intervention was effective, modeling individual trajectories may help our understanding of mechanisms in the pathways of change. Larger, more diverse samples and microrandomized or N-of-1 designs are now required to delineate dose-by-timing interactions and to guide the optimal tailoring, scalable implementation, and equitable use of digital interventions to improve youth mental health.

## Supplementary material

10.2196/77150 Multimedia Appendix 1 Supplementary information on deviations from the preregistration, exploratory analyses of same-day post-task associations, further information on exploratory analyses, crude models, supplementary exploratory findings, findings from the preregistered analyses, and findings from the preregistered analyses.

10.2196/77150 Checklist 1 CONSORT-eHEALTH checklist (V 1.6.1).

## Abbreviations

CFT: compassion-focused therapy
CHARMS: clinical high at-risk mental state
CONSORT: Consolidated Standards of Reporting Trials
CONSORT-EHEALTH: Consolidated Standards of Reporting Trials of Electronic and Mobile Health Applications and Online Telehealth
EMA: ecological momentary assessment
EMI: ecological momentary intervention
RCT: randomized controlled trial
