# Supplementary material for: Proximal Effects of a Transdiagnostic, Ecological Momentary Intervention for Enhancing Resilience in Help-Seeking Young People (EMIcompass): Findings From a Secondary Analysis of an Exploratory Randomized Controlled Trial
Source: J Med Internet Res. 2026 Sep 3;28:e77150. doi: 10.2196/77150 (PMC13543658; doi:10.2196/77150)
Supplement: Supplementary file 1 [file jmir-v28-e77150-s002.pdf]

CONSORT-EHEALTH (V 1.6.1) - Submission/Publication Form

The CONSORT-EHEALTH checklist is intended for authors of randomized trials evaluating web-based and Internet-based applications/interventions, including mobile interventions, electronic games (incl multiplayer games), social media, certain telehealth applications, and other interactive and/or networked electronic applications. Some of the items (e.g. all subitems under item 5 - description of the intervention) may also be applicable for other study designs.

The goal of the CONSORT EHEALTH checklist and guideline is to be
a) a guide for reporting for authors of RCTs,
b) to form a basis for appraisal of an ehealth trial (in terms of validity)

CONSORT-EHEALTH items/subitems are MANDATORY reporting items for studies published in the Journal of Medical Internet Research and other journals / scientific societies endorsing the checklist.

Items numbered 1., 2., 3., 4a., 4b etc are original CONSORT or CONSORT-NPT (non-pharmacologic treatment) items.
Items with Roman numerals (i., ii, iii, iv etc.) are CONSORT-EHEALTH extensions/clarifications.

As the CONSORT-EHEALTH checklist is still considered in a formative stage, we would ask that you also RATE ON A SCALE OF 1-5 how important/useful you feel each item is FOR THE PURPOSE OF THE CHECKLIST and reporting guideline (optional).

Mandatory reporting items are marked with a red *.
In the textboxes, either copy & paste the relevant sections from your manuscript into this form - please include any quotes from your manuscript in QUOTATION MARKS,
or answer directly by providing additional information not in the manuscript, or elaborating on why the item was not relevant for this study.

YOUR ANSWERS WILL BE PUBLISHED AS A SUPPLEMENTARY FILE TO YOUR PUBLICATION IN JMIR AND ARE CONSIDERED PART OF YOUR PUBLICATION (IF ACCEPTED).
Please fill in these questions diligently. Information will not be copyedited, so please use proper spelling and grammar, use correct capitalization, and avoid abbreviations.

DO NOT FORGET TO SAVE AS PDF _AND_ CLICK THE SUBMIT BUTTON SO YOUR ANSWERS ARE IN OUR DATABASE !!!

Citation Suggestion (if you append the pdf as Appendix we suggest to cite this paper in the caption):
Eysenbach G, CONSORT-EHEALTH Group
CONSORT-EHEALTH: Improving and Standardizing Evaluation Reports of Web-based and Mobile Health Interventions
J Med Internet Res 2011;13(4):e126

URL: http://www.jmir.org/2011/4/e126/
doi: 10.2196/jmir.1923
PMID: 22209829

gugel.jessica@googlemail.com Konto wechseln

Nicht freigegeben

Wird gespeichert…

* Gibt eine erforderliche Frage an

Your name *

First Last

Jessica Gugel

Primary Affiliation (short), City, Country *

University of Toronto, Toronto, Canada

Central Institute of Mental Health, Mannheim, (

Your e-mail address *

abc@gmail.com

Jessica.Gugel@zi-mannheim.de

Title of your manuscript *

Provide the (draft) title of your manuscript.

Proximal Effects of a Transdiagnostic, Ecological Momentary Intervention for Enhancing Resilience in Help-Seeking Young People (EMIcompass): Findings from a Secondary Analysis of an Exploratory Randomized Controlled Trial

Name of your App/Software/Intervention *

If there is a short and a long/alternate name, write the short name first and add the long name in brackets.

EMIcompass

Evaluated Version (if any)

e.g. "V1", "Release 2017-03-01", "Version 2.0.27913"

Release 2019-08-06

Language(s) *

What language is the intervention/app in? If multiple languages are available, separate by comma (e.g. "English, French")

German

URL of your Intervention Website or App

e.g. a direct link to the mobile app on app in appstore (itunes, Google Play), or URL of the website. If the intervention is a DVD or hardware, you can also link to an Amazon page.

https://play.google.com/store/apps/details?id=com.movisens.xs.android.core&hl=de&pli=1

URL of an image/screenshot (optional)

Meine Antwort

Accessibility *

Can an enduser access the intervention presently?

- ○ access is free and open
- ◉ access only for special usergroups, not open
- ○ access is open to everyone, but requires payment/subscription/in-app purchases
- ○ app/intervention no longer accessible
- ○ Sonstiges:

Primary Medical Indication/Disease/Condition *

e.g. "Stress", "Diabetes", or define the target group in brackets after the condition, e.g. "Autism (Parents of children with)", "Alzheimers (Informal Caregivers of)"

No medical indication. Target group: youth age

Primary Outcomes measured in trial *

comma-separated list of primary outcomes reported in the trial

In the current secondary analysis, the followin

Secondary/other outcomes

Are there any other outcomes the intervention is expected to affect?

Meine Antwort

Recommended "Dose" *

What do the instructions for users say on how often the app should be used?

- (●) Approximately Daily
- ( ) Approximately Weekly
- ( ) Approximately Monthly
- ( ) Approximately Yearly
- ( ) "as needed"
- ( ) Sonstiges:

Approx. Percentage of Users (starters) still using the app as recommended after 3 months *

- (●) unknown / not evaluated
- ( ) 0-10%
- ( ) 11-20%
- ( ) 21-30%
- ( ) 31-40%
- ( ) 41-50%
- ( ) 51-60%
- ( ) 61-70%
- ( ) 71%-80%
- ( ) 81-90%
- ( ) 91-100%
- ( ) Sonstiges:

Overall, was the app/intervention effective? *

- ( ) yes: all primary outcomes were significantly better in intervention group vs control
- ( ) partly: SOME primary outcomes were significantly better in intervention group vs control
- ( ) no statistically significant difference between control and intervention
- ( ) potentially harmful: control was significantly better than intervention in one or more outcomes
- ( ) inconclusive: more research is needed
- (x) Sonstiges: The current analysis is no RCT, but a secondary analysis of an exi

Article Preparation Status/Stage *

At which stage in your article preparation are you currently (at the time you fill in this form)

- ( ) not submitted yet - in early draft status
- ( ) not submitted yet - in late draft status, just before submission
- ( ) submitted to a journal but not reviewed yet
- (x) submitted to a journal and after receiving initial reviewer comments
- ( ) submitted to a journal and accepted, but not published yet
- ( ) published
- ( ) Sonstiges:

Journal *

If you already know where you will submit this paper (or if it is already submitted), please provide the journal name (if it is not JMIR, provide the journal name under "other")

- ○ not submitted yet / unclear where I will submit this
- ◉ Journal of Medical Internet Research (JMIR)
- ○ JMIR mHealth and UHealth
- ○ JMIR Serious Games
- ○ JMIR Mental Health
- ○ JMIR Public Health
- ○ JMIR Formative Research
- ○ Other JMIR sister journal
- ○ Sonstiges:

Is this a full powered effectiveness trial or a pilot/feasibility trial? *

- ◉ Pilot/feasibility
- ○ Fully powered

Manuscript tracking number *

If this is a JMIR submission, please provide the manuscript tracking number under "other" (The ms tracking number can be found in the submission acknowledgement email, or when you login as author in JMIR. If the paper is already published in JMIR, then the ms tracking number is the four-digit number at the end of the DOI, to be found at the bottom of each published article in JMIR)

- ○ no ms number (yet) / not (yet) submitted to / published in JMIR
- ◉ Sonstiges: 77150

TITLE AND ABSTRACT

1a) TITLE: Identification as a randomized trial in the title

1a) Does your paper address CONSORT item 1a? *

I.e does the title contain the phrase "Randomized Controlled Trial"? (if not, explain the reason under "other")

- ○ yes
- ◉ Sonstiges: it contains the phrase "Findings from a Secondary Analysis of an I

1a-i) Identify the mode of delivery in the title

Identify the mode of delivery. Preferably use “web-based” and/or “mobile” and/or “electronic game” in the title. Avoid ambiguous terms like “online”, “virtual”, “interactive”. Use “Internet-based” only if Intervention includes non-web-based Internet components (e.g. email), use “computer-based” or “electronic” only if offline products are used. Use “virtual” only in the context of “virtual reality” (3-D worlds). Use “online” only in the context of “online support groups”. Complement or substitute product names with broader terms for the class of products (such as “mobile” or “smart phone” instead of “iphone”), especially if the application runs on different platforms.

	1	2	3	4	5	
subitem not at all important	○	○	○	○	○	essential

Does your paper address subitem 1a-i? *

Copy and paste relevant sections from manuscript title (include quotes in quotation marks "like this" to indicate direct quotes from your manuscript), or elaborate on this item by providing additional information not in the ms, or briefly explain why the item is not applicable/relevant for your study

"Ecological Momentary Intervention"

1a-ii) Non-web-based components or important co-interventions in title

Mention non-web-based components or important co-interventions in title, if any (e.g., “with telephone support”).

	1	2	3	4	5	
subitem not at all important	○	○	○	○	○	essential

Does your paper address subitem 1a-ii?

Copy and paste relevant sections from manuscript title (include quotes in quotation marks "like this" to indicate direct quotes from your manuscript), or elaborate on this item by providing additional information not in the ms, or briefly explain why the item is not applicable/relevant for your study

Hybrid intervention, information in abstract: "EMIcompass is a novel, transdiagnostic, hybrid, compassion-focused EMI for prevention and early intervention of severe mental health conditions in help-seeking youth." information in the manuscript: "The EMIcompass intervention consisted of a 6-week compassion-focused EMI and 4 biweekly sessions (3 training sessions and one review session) as well as an optional on-demand session with a duration of 45-60 minutes."

1a-iii) Primary condition or target group in the title

Mention primary condition or target group in the title, if any (e.g., “for children with Type I Diabetes”) Example: A Web-based and Mobile Intervention with Telephone Support for Children with Type I Diabetes: Randomized Controlled Trial

	1	2	3	4	5	
subitem not at all important	○	○	○	○	○	essential

Does your paper address subitem 1a-iii? *

Copy and paste relevant sections from manuscript title (include quotes in quotation marks "like this" to indicate direct quotes from your manuscript), or elaborate on this item by providing additional information not in the ms, or briefly explain why the item is not applicable/relevant for your study

"Help-Seeking Young People"

1b) ABSTRACT: Structured summary of trial design, methods, results, and conclusions

NPT extension: Description of experimental treatment, comparator, care providers, centers, and blinding status.

1b-i) Key features/functionalities/components of the intervention and comparator in the METHODS section of the ABSTRACT

Mention key features/functionalities/components of the intervention and comparator in the abstract. If possible, also mention theories and principles used for designing the site. Keep in mind the needs of systematic reviewers and indexers by including important synonyms. (Note: Only report in the abstract what the main paper is reporting. If this information is missing from the main body of text, consider adding it)

	1	2	3	4	5	
subitem not at all important	○	○	○	○	○	essential

Does your paper address subitem 1b-i? *

Copy and paste relevant sections from the manuscript abstract (include quotes in quotation marks "like this" to indicate direct quotes from your manuscript), or elaborate on this item by providing additional information not in the ms, or briefly explain why the item is not applicable/relevant for your study

"EMIcompass is a novel, transdiagnostic, hybrid, compassion-focused EMI for prevention and early intervention of severe mental health conditions in help-seeking youth."; "We used within-subject data collected as part of the EMIcompass intervention in the experimental condition, in which participants were offered EMI tasks and voluntarily decided whether or not to complete them."

1b-ii) Level of human involvement in the METHODS section of the ABSTRACT

Clarify the level of human involvement in the abstract, e.g., use phrases like “fully automated” vs. “therapist/nurse/care provider/physician-assisted” (mention number and expertise of providers involved, if any). (Note: Only report in the abstract what the main paper is reporting. If this information is missing from the main body of text, consider adding it)

	1	2	3	4	5	
subitem not at all important	○	○	○	○	○	essential

Does your paper address subitem 1b-ii?

Copy and paste relevant sections from the manuscript abstract (include quotes in quotation marks "like this" to indicate direct quotes from your manuscript), or elaborate on this item by providing additional information not in the ms, or briefly explain why the item is not applicable/relevant for your study

"EMIcompass is a novel, transdiagnostic, hybrid, compassion-focused EMI for prevention and early intervention of severe mental health conditions in help-seeking youth. Data for our secondary analysis were derived from the experimental condition of the EMIcompass exploratory randomized controlled trial, in which youth aged 14-25 with early mental health problems were randomly allocated to the EMIcompass intervention + TAU (experimental condition) or TAU only (control condition)."

1b-iii) Open vs. closed, web-based (self-assessment) vs. face-to-face assessments in the METHODS section of the ABSTRACT

Mention how participants were recruited (online vs. offline), e.g., from an open access website or from a clinic or a closed online user group (closed usergroup trial), and clarify if this was a purely web-based trial, or there were face-to-face components (as part of the intervention or for assessment). Clearly say if outcomes were self-assessed through questionnaires (as common in web-based trials). Note: In traditional offline trials, an open trial (open-label trial) is a type of clinical trial in which both the researchers and participants know which treatment is being administered. To avoid confusion, use “blinded” or “unblinded” to indicated the level of blinding instead of “open”, as “open” in web-based trials usually refers to “open access” (i.e. participants can self-enrol). (Note: Only report in the abstract what the main paper is reporting. If this information is missing from the main body of text, consider adding it)

	1	2	3	4	5	
subitem not at all important	○	○	○	○	○	essential

Does your paper address subitem 1b-iii?

Copy and paste relevant sections from the manuscript abstract (include quotes in quotation marks "like this" to indicate direct quotes from your manuscript), or elaborate on this item by providing additional information not in the ms, or briefly explain why the item is not applicable/relevant for your study

We used self-reported within-subject EMA data collected as part of the EMIcompass intervention in the experimental condition, in which participants were offered EMI tasks and voluntarily decided whether or not to complete them."

1b-iv) RESULTS section in abstract must contain use data

Report number of participants enrolled/assessed in each group, the use/uptake of the intervention (e.g., attrition/adherence metrics, use over time, number of logins etc.), in addition to primary/secondary outcomes. (Note: Only report in the abstract what the main paper is reporting. If this information is missing from the main body of text, consider adding it)

	1	2	3	4	5	
subitem not at all important	○	○	○	○	○	essential

Does your paper address subitem 1b-iv?

Copy and paste relevant sections from the manuscript abstract (include quotes in quotation marks "like this" to indicate direct quotes from your manuscript), or elaborate on this item by providing additional information not in the ms, or briefly explain why the item is not applicable/relevant for your study

"Data on proximal effects of EMI interactive task completion on negative affect, stress, and stress reactivity at the subsequent EMA were available in a small subsample of n=37 participants." Only participants from the experimental condition of the existing exploratory RCT were included, thus there are no groups.

1b-v) CONCLUSIONS/DISCUSSION in abstract for negative trials

Conclusions/Discussions in abstract for negative trials: Discuss the primary outcome - if the trial is negative (primary outcome not changed), and the intervention was not used, discuss whether negative results are attributable to lack of uptake and discuss reasons. (Note: Only report in the abstract what the main paper is reporting. If this information is missing from the main body of text, consider adding it)

	1	2	3	4	5	
subitem not at all important	○	○	○	○	○	essential

Does your paper address subitem 1b-v?

Copy and paste relevant sections from the manuscript abstract (include quotes in quotation marks "like this" to indicate direct quotes from your manuscript), or elaborate on this item by providing additional information not in the ms, or briefly explain why the item is not applicable/relevant for your study

"This secondary analysis implemented a novel approach to advancing our understanding of proximal effects within an EMI framework. While our findings illustrate the feasibility of investigating micro-level processes within an EMI framework, they remain equivocal with regard to proximal effects, owing to limited power in this small sample. Further research with larger samples and appropriate study designs such as micro-randomized or N-of-1 designs is needed to clarify how EMI components impact momentary mental health mechanisms and outcomes."

INTRODUCTION

2a) In INTRODUCTION: Scientific background and explanation of rationale

2a-i) Problem and the type of system/solution

Describe the problem and the type of system/solution that is object of the study: intended as stand-alone intervention vs. incorporated in broader health care program? Intended for a particular patient population? Goals of the intervention, e.g., being more cost-effective to other interventions, replace or complement other solutions? (Note: Details about the intervention are provided in “Methods” under 5)

	1	2	3	4	5	
subitem not at all important	○	○	○	○	○	essential

Does your paper address subitem 2a-i? *

Copy and paste relevant sections from the manuscript (include quotes in quotation marks "like this" to indicate direct quotes from your manuscript), or elaborate on this item by providing additional information not in the ms, or briefly explain why the item is not applicable/relevant for your study

"However, more research is needed in order to elucidate mechanisms of change to inform the development of targeted innovations with the potential to enhance intervention effects [20]. "

"While previous research shows that compassion-focused interventions may have proximal effects on mental health mechanisms and outcomes in laboratory settings [45, 46], to our knowledge, there is currently no evidence of proximal effects on negative affect, stress, and stress reactivity in real-life settings. Examining proximal effects may be crucial to improve existing and develop new EMIs in the future."

"However, evidence on the feasibility of such a micro-level approach and initial signals of proximal effects of EMI completion on putative momentary mechanisms and outcomes is pending."

2a-ii) Scientific background, rationale: What is known about the (type of) system

Scientific background, rationale: What is known about the (type of) system that is the object of the study (be sure to discuss the use of similar systems for other conditions/diagnoses, if appropiate), motivation for the study, i.e. what are the reasons for and what is the context for this specific study, from which stakeholder viewpoint is the study performed, potential impact of findings [2]. Briefly justify the choice of the comparator.

	1	2	3	4	5	
subitem not at all important	○	○	○	○	○	essential

Does your paper address subitem 2a-ii? *

Copy and paste relevant sections from the manuscript (include quotes in quotation marks "like this" to indicate direct quotes from your manuscript), or elaborate on this item by providing additional information not in the ms, or briefly explain why the item is not applicable/relevant for your study

"EMA can be implemented through dedicated smartphone applications in the form of structured digital diary techniques, allowing users to complete brief questionnaires about their symptoms, mood, and context at specific moments [8]. EMA, thereby, provides intensive longitudinal data on experience and behavior and their fluctuations in the context of everyday life, minimizing the risk of biases, including recall bias [9-11]"

"Furthermore, EMA principles and techniques can be used to inform digital mental health interventions. Ecological momentary interventions (EMIs) represent an innovative approach, enabling real-time delivery of intervention components tailored to person, moment, and context based on EMA data and the use of interactive/adaptive delivery schemes [12-15]."

"EMIs can be used to specifically target putative mechanisms that have been linked to the development and persistence of mental health conditions [4]. Possible mechanisms include emotion regulation [21, 22] and stress reactivity, referring to an increased negative affect in response to minor stressors in daily life [23-25]."

"Individuals at risk for various mental health conditions, including depression and psychosis, have been found to have an increased stress reactivity [23, 24, 26-30]."

2b) In INTRODUCTION: Specific objectives or hypotheses

Does your paper address CONSORT subitem 2b? *

Copy and paste relevant sections from the manuscript (include quotes in quotation marks "like this" to indicate direct quotes from your manuscript), or elaborate on this item by providing additional information not in the ms, or briefly explain why the item is not applicable/relevant for your study

"Against this background, the aim of the current study was to explore proximal effects of the EMIcompass intervention at the micro-level, i.e., proximal effects of completing EMI components on momentary mental health mechanisms and outcomes in youth with early mental health problems. We posited that for participants in the experimental condition of the EMIcompass trial, momentary negative affect, stress, and stress reactivity at the subsequent EMA would be lower at observations following the completion of EMI interactive tasks, than compared to non-completion (when controlled for momentary negative affect, stress, and stress reactivity at the preceding EMA). The following exploratory hypotheses were tested (focusing on measures of effect size):

H1: Momentary negative affect will, on average, be lower at the subsequent EMA after EMI interactive task completion when compared to task non-completion in moments of high stress or negative affect, when controlled for momentary negative affect at the preceding EMA, time between EMAs, clinical staging, age, gender, context change between EMAs and task time.

H2: Momentary stress will, on average, be lower at the subsequent EMA after EMI interactive task completion when compared to task non-completion in moments of high stress or negative affect, when controlled for momentary stress at the preceding EMA, time between EMAs, clinical staging, age, gender, context change between EMAs and task time.

H3: Momentary stress reactivity will, on average, be lower at the subsequent EMA after EMI interactive task completion when compared to task non-completion in moments of high stress or negative affect when controlled for momentary stress reactivity at the preceding EMA, time between EMAs, clinical staging, age, gender, context change between EMAs and task time."

METHODS

3a) Description of trial design (such as parallel, factorial) including allocation ratio

Does your paper address CONSORT subitem 3a? *

Copy and paste relevant sections from the manuscript (include quotes in quotation marks "like this" to indicate direct quotes from your manuscript), or elaborate on this item by providing additional information not in the ms, or briefly explain why the item is not applicable/relevant for your study

This is a secondary analysis of an existing exploratory RCT,. The study design of the exploratory RCT and the current analysis can be found in the section Study Design.

3b) Important changes to methods after trial commencement (such as eligibility criteria), with reasons

Does your paper address CONSORT subitem 3b? *

Copy and paste relevant sections from the manuscript (include quotes in quotation marks "like this" to indicate direct quotes from your manuscript), or elaborate on this item by providing additional information not in the ms, or briefly explain why the item is not applicable/relevant for your study

All changes in the analyses that deviated from the preregistration are described in Supplement 1.

3b-i) Bug fixes, Downtimes, Content Changes

Bug fixes, Downtimes, Content Changes: ehealth systems are often dynamic systems. A description of changes to methods therefore also includes important changes made on the intervention or comparator during the trial (e.g., major bug fixes or changes in the functionality or content) (5-iii) and other “unexpected events” that may have influenced study design such as staff changes, system failures/downtimes, etc. [2].

	1	2	3	4	5	
subitem not at all important	○	○	○	○	○	essential

Does your paper address subitem 3b-i?

Copy and paste relevant sections from the manuscript (include quotes in quotation marks "like this" to indicate direct quotes from your manuscript), or elaborate on this item by providing additional information not in the ms, or briefly explain why the item is not applicable/relevant for your study

No bug fixes were neccessary. However, the exploratory RCT, from which we use data for our secondary analysis, was affected by the COVID-19 pandemic.

"Last, the EMIcompass trial was conducted and EMA data collected during the COVID-19 pandemic, which might have altered participants' daily lives through changes like restricted social contacts, heightened health-related stress, and a shift from university or work to home-office settings [121, 122]."

4a) Eligibility criteria for participants

Does your paper address CONSORT subitem 4a? *

Copy and paste relevant sections from the manuscript (include quotes in quotation marks "like this" to indicate direct quotes from your manuscript), or elaborate on this item by providing additional information not in the ms, or briefly explain why the item is not applicable/relevant for your study

"The inclusion criteria were: 1) aged 14-25 years, 2) meeting criteria for stage 1a (k10 score≥20 [49, 50]), stage 1b (CHARMS), or stage 2 (first episode of psychotic, bipolar, severe depressive, or severe anxiety disorder) in accordance with the clinical staging model by Hartmann et al. ([47], high stress reactivity assessed with a 2-item self-report measure or the CAAARMS subscale on impaired tolerance to everyday stress [51], 4) reduced positive affect or increased negative affect based on normative scores [52] of the Positive and Negative Affect Scale [53], 5) willingness to participate, and 6) ability to provide written informed consent (or consent by caregivers or legal guardians for minors). The exclusion criteria were: primary diagnosis of alcohol/substance abuse/dependence (SCID-5[54]), 2) symptoms precipitated by an organic disease, 3) insufficient language proficiency in German, 4) diagnoses of a learning disability, and 5) acute suicidality (CAARMS score > 4 [51])."

4a-i) Computer / Internet literacy

Computer / Internet literacy is often an implicit “de facto” eligibility criterion - this should be explicitly clarified.

	1	2	3	4	5	
subitem not at all important	○	○	○	○	○	essential

Does your paper address subitem 4a-i?

Copy and paste relevant sections from the manuscript (include quotes in quotation marks "like this" to indicate direct quotes from your manuscript), or elaborate on this item by providing additional information not in the ms, or briefly explain why the item is not applicable/relevant for your study

Since this is a secondary analysis using data from an existing exploratory RCT, no changes in the eligibility criteria could be made. For consistency across papers using data from this exploratory RCT, we did not change the wording. Internet literacy was not specifically named, however, relevant components for this are listed:

"The exclusion criteria were: [...] 2) symptoms precipitated by an organic disease, 3) insufficient language proficiency in German, 4) diagnoses of a learning disability [...]).

4a-ii) Open vs. closed, web-based vs. face-to-face assessments:

Open vs. closed, web-based vs. face-to-face assessments: Mention how participants were recruited (online vs. offline), e.g., from an open access website or from a clinic, and clarify if this was a purely web-based trial, or there were face-to-face components (as part of the intervention or for assessment), i.e., to what degree got the study team to know the participant. In online-only trials, clarify if participants were quasi-anonymous and whether having multiple identities was possible or whether technical or logistical measures (e.g., cookies, email confirmation, phone calls) were used to detect/prevent these.

	1	2	3	4	5	
subitem not at all important	○	○	○	○	○	essential

Does your paper address subitem 4a-ii? *

Copy and paste relevant sections from the manuscript (include quotes in quotation marks "like this" to indicate direct quotes from your manuscript), or elaborate on this item by providing additional information not in the ms, or briefly explain why the item is not applicable/relevant for your study

"In the EMIcompass RCT, youth with early mental health problems aged 14-25 years were recruited from mental health services at the Central Institute of Mental Health in Mannheim, Germany as well as local registries, online and social media advertisements."

"The 'EMIcompass' intervention, a transdiagnostic, compassion-focused, hybrid ecological momentary intervention for improving resilience in youth with early mental health problems, was specifically designed building on previous findings on EMA and EMI [4, 14, 32] as well as transdiagnostic momentary mechanisms [13, 24, 33-35] to target psychological distress, clinical symptoms, and candidate mechanisms [36, 37]."

"The EMIcompass intervention consisted of a 6-week compassion-focused EMI and 4 biweekly sessions (3 training sessions and one review session) as well as an optional on-demand session with a duration of 45-60 minutes. All sessions were delivered to the participants individually, either face-to-face or using a certified and encrypted video conferencing system."

4a-iii) Information giving during recruitment

Information given during recruitment. Specify how participants were briefed for recruitment and in the informed consent procedures (e.g., publish the informed consent documentation as appendix, see also item X26), as this information may have an effect on user self-selection, user expectation and may also bias results.

	1	2	3	4	5	
subitem not at all important	○	○	○	○	○	essential

Does your paper address subitem 4a-iii?

Copy and paste relevant sections from the manuscript (include quotes in quotation marks "like this" to indicate direct quotes from your manuscript), or elaborate on this item by providing additional information not in the ms, or briefly explain why the item is not applicable/relevant for your study

"To minimize social desirability bias, all participants were instructed in detail before and during the EMA assessment."

4b) Settings and locations where the data were collected

Does your paper address CONSORT subitem 4b? *

Copy and paste relevant sections from the manuscript (include quotes in quotation marks "like this" to indicate direct quotes from your manuscript), or elaborate on this item by providing additional information not in the ms, or briefly explain why the item is not applicable/relevant for your study

"In the EMIcompass RCT, youth with early mental health problems aged 14-25 years were recruited from mental health services at the Central Institute of Mental Health in Mannheim, Germany as well as local registries, online and social media advertisements."

4b-i) Report if outcomes were (self-)assessed through online questionnaires

Clearly report if outcomes were (self-)assessed through online questionnaires (as common in web-based trials) or otherwise.

	1	2	3	4	5	
subitem not at all important	○	○	○	○	○	essential

Does your paper address subitem 4b-i? *

Copy and paste relevant sections from the manuscript (include quotes in quotation marks "like this" to indicate direct quotes from your manuscript), or elaborate on this item by providing additional information not in the ms, or briefly explain why the item is not applicable/relevant for your study

"Self-reported age and gender (i.e., female, male, non-binary) were assessed and clinical staging (i.e., current psychological distress, clinical high at-risk mental state, or first treated episode of severe mental disorder) was rated by blinded assessors based on standardized interviews, self-report, and observer ratings at baseline."

"EMA data of momentary affect and stress was assessed six times per day, on 3 consecutive days per week during the intervention period."

"Participants were asked to report the pleasantness of the most important event that has happened since the last EMA on a bipolar 7-point Likert scale ranging from -3 (= very unpleasant) to +3 (= very pleasant) as well as the pleasantness of the activity they performed just before the EMA and the pleasantness of their current social contact on 7-point Likert scales ranging from 1 (= very unpleasant) to 7 (= very pleasant). Additionally, they were asked to answer the questions "I'm taking care of myself / I'm taking care of somebody" and "I would prefer to be alone / I would prefer to have company" (1=not at all, 7=very much) as follow-up questions to "Who am I with?"."

"During the EMI period, passive usage data, including date and time when a task was delivered, the delivery scheme (i.e., enhancing, consolidating, interactive), and the task type (i.e., self-compassionate writing, experiencing emotions as a wave, discovering their own compassionate self, imagining a compassionate companion, counting their breath, breathing with breaks, finding a calming colour, writing a compassionate message, imagining a safe and calm place, and emotional compass) were recorded automatically."

4b-ii) Report how institutional affiliations are displayed

Report how institutional affiliations are displayed to potential participants [on ehealth media], as affiliations with prestigious hospitals or universities may affect volunteer rates, use, and reactions with regards to an intervention.(Not a required item – describe only if this may bias results)

	1	2	3	4	5	
subitem not at all important	○	○	○	○	○	essential

Does your paper address subitem 4b-ii?

Copy and paste relevant sections from the manuscript (include quotes in quotation marks "like this" to indicate direct quotes from your manuscript), or elaborate on this item by providing additional information not in the ms, or briefly explain why the item is not applicable/relevant for your study

The insitution involved in EMIcompass (Central Institute of Mental Health) was mentioned with its logo on the webpage for the intervention. Participants were informed about the institutional affiliation during informed consent. We do not believe that this biased the results and therefore do not report this optional item in the manuscript.

5) The interventions for each group with sufficient details to allow replication, including how and when they were actually administered

5-i) Mention names, credential, affiliations of the developers, sponsors, and owners

Mention names, credential, affiliations of the developers, sponsors, and owners [6] (if authors/evaluators are owners or developer of the software, this needs to be declared in a “Conflict of interest” section or mentioned elsewhere in the manuscript).

	1	2	3	4	5	
subitem not at all important	○	○	○	○	○	essential

Does your paper address subitem 5-i?

Copy and paste relevant sections from the manuscript (include quotes in quotation marks "like this" to indicate direct quotes from your manuscript), or elaborate on this item by providing additional information not in the ms, or briefly explain why the item is not applicable/relevant for your study

"The ‘EMIcompass’ intervention, a transdiagnostic, compassion-focused, hybrid ecological momentary intervention for improving resilience in youth with early mental health problems, was specifically designed building on previous findings on EMA and EMI [4, 14, 32] as well as transdiagnostic momentary mechanisms [13, 24, 33-35] to target psychological distress, clinical symptoms, and candidate mechanisms [36, 37]."

"A structured manual was developed based on existing manuals, expert consultation, and clinical experience, which guided the selection and sequencing of intervention elements (for further information, see [38])."

5-ii) Describe the history/development process

Describe the history/development process of the application and previous formative evaluations (e.g., focus groups, usability testing), as these will have an impact on adoption/use rates and help with interpreting results.

	1	2	3	4	5	
subitem not at all important	○	○	○	○	○	essential

Does your paper address subitem 5-ii?

Copy and paste relevant sections from the manuscript (include quotes in quotation marks "like this" to indicate direct quotes from your manuscript), or elaborate on this item by providing additional information not in the ms, or briefly explain why the item is not applicable/relevant for your study

"The 'EMIcompass' intervention, a transdiagnostic, compassion-focused, hybrid ecological momentary intervention for improving resilience in youth with early mental health problems, was specifically designed building on previous findings on EMA and EMI [4, 14, 32] as well as transdiagnostic momentary mechanisms [13, 24, 33-35] to target psychological distress, clinical symptoms, and candidate mechanisms [36, 37]."

"A structured manual was developed based on existing manuals, expert consultation, and clinical experience, which guided the selection and sequencing of intervention elements (for further information, see [38])."

The exploratory RCT, from which we used data for our current secondary anaylsis, was named as exploratory anaysis to demonstrate feasability of trial methodology and intervention delivery: "In an exploratory parallel-group, assessor-blind randomized controlled trial (RCT), first evidence of feasibility of trial methodology and intervention delivery as well as initial signals of efficacy on candidate mechanisms (i.e., emotional reactivity, stress reactivity, threat anticipation, resilience, and aberrant salience) and outcomes (i.e., quality of life and BPRS total scores) of EMIcompass were observed [36]."

5-iii) Revisions and updating

Revisions and updating. Clearly mention the date and/or version number of the application/intervention (and comparator, if applicable) evaluated, or describe whether the intervention underwent major changes during the evaluation process, or whether the development and/or content was “frozen” during the trial. Describe dynamic components such as news feeds or changing content which may have an impact on the replicability of the intervention (for unexpected events see item 3b).

	1	2	3	4	5	
subitem not at all important	○	○	○	○	○	essential

Does your paper address subitem 5-iii?

Copy and paste relevant sections from the manuscript (include quotes in quotation marks "like this" to indicate direct quotes from your manuscript), or elaborate on this item by providing additional information not in the ms, or briefly explain why the item is not applicable/relevant for your study

Not applicable, there were no changes during the intervention period, so the initial description of the intervention was always accurate.

5-iv) Quality assurance methods

Provide information on quality assurance methods to ensure accuracy and quality of information provided [1], if applicable.

	1	2	3	4	5	
subitem not at all important	○	○	○	○	○	essential

Does your paper address subitem 5-iv?

Copy and paste relevant sections from the manuscript (include quotes in quotation marks "like this" to indicate direct quotes from your manuscript), or elaborate on this item by providing additional information not in the ms, or briefly explain why the item is not applicable/relevant for your study

"To minimize social desirability bias, all participants were instructed in detail before and during the EMA assessment."

"Based on the trained psychologists' impression in the sessions and the participants' experiences with the intervention tasks in the first 2 weeks of the intervention, participants were allocated to the basic or the elaborate track of the intervention [38] (for further information, see Supplement 3)."

5-v) Ensure replicability by publishing the source code, and/or providing screenshots/screen-capture video, and/or providing flowcharts of the algorithms used

Ensure replicability by publishing the source code, and/or providing screenshots/screen-capture video, and/or providing flowcharts of the algorithms used. Replicability (i.e., other researchers should in principle be able to replicate the study) is a hallmark of scientific reporting.

	1	2	3	4	5	
subitem not at all important	○	○	○	○	○	essential

Does your paper address subitem 5-v?

Copy and paste relevant sections from the manuscript (include quotes in quotation marks "like this" to indicate direct quotes from your manuscript), or elaborate on this item by providing additional information not in the ms, or briefly explain why the item is not applicable/relevant for your study

Due to movisens GmbH being the owner of the platform, the source code of the app can not be provided.

5-vi) Digital preservation

Digital preservation: Provide the URL of the application, but as the intervention is likely to change or disappear over the course of the years; also make sure the intervention is archived (Internet Archive, webcitation.org, and/or publishing the source code or screenshots/videos alongside the article). As pages behind login screens cannot be archived, consider creating demo pages which are accessible without login.

	1	2	3	4	5	
subitem not at all important	○	○	○	○	○	essential

Does your paper address subitem 5-vi?

Copy and paste relevant sections from the manuscript (include quotes in quotation marks "like this" to indicate direct quotes from your manuscript), or elaborate on this item by providing additional information not in the ms, or briefly explain why the item is not applicable/relevant for your study

The app movisensXS is accessible via the Google Playstore (https://play.google.com/store/apps/details?id=com.movisens.xs.android.core&hl=de&pli=1), but the EMIcompass intervention is not publicly available.

5-vii) Access

Access: Describe how participants accessed the application, in what setting/context, if they had to pay (or were paid) or not, whether they had to be a member of specific group. If known, describe how participants obtained "access to the platform and Internet" [1]. To ensure access for editors/reviewers/readers, consider to provide a "backdoor" login account or demo mode for reviewers/readers to explore the application (also important for archiving purposes, see vi).

	1	2	3	4	5	
subitem not at all important	○	○	○	○	○	essential

Does your paper address subitem 5-vii? *

Copy and paste relevant sections from the manuscript (include quotes in quotation marks "like this" to indicate direct quotes from your manuscript), or elaborate on this item by providing additional information not in the ms, or briefly explain why the item is not applicable/relevant for your study

The intervention was only available to participants during the intervention period.

"The smartphone-based EMI was administered through a smartphone-based app (movisensXS, movisens GmbH, Karlsruhe, Germany) on study devices and translated the training from the intervention sessions into individuals' daily lives [38]."

"After the intervention period, participants were asked to return the study devices and no longer had access to the app."

5-viii) Mode of delivery, features/functionalities/components of the intervention and comparator, and the theoretical framework

Describe mode of delivery, features/functionalities/components of the intervention and comparator, and the theoretical framework [6] used to design them (instructional strategy [1], behaviour change techniques, persuasive features, etc., see e.g., [7, 8] for terminology). This includes an in-depth description of the content (including where it is coming from and who developed it) [1]," whether [and how] it is tailored to individual circumstances and allows users to track their progress and receive feedback" [6]. This also includes a description of communication delivery channels and – if computer-mediated communication is a component – whether communication was synchronous or asynchronous [6]. It also includes information on presentation strategies [1], including page design principles, average amount of text on pages, presence of hyperlinks to other resources, etc. [1].

	1	2	3	4	5	
subitem not at all important	○	○	○	○	○	essential

Does your paper address subitem 5-viii? *

Copy and paste relevant sections from the manuscript (include quotes in quotation marks "like this" to indicate direct quotes from your manuscript), or elaborate on this item by providing additional information not in the ms, or briefly explain why the item is not applicable/relevant for your study

"EMIcompass followed a compassion-focused approach [40, 41], in which participants were offered breathing exercises, soothing imagery, and self-compassionate imagery and writing techniques. The smartphone-based EMI translated the training from the intervention sessions into individuals' daily lives [38]. Based on the trained psychologists' impression in the sessions and the participants' experiences with the intervention tasks in the first 2 weeks of the intervention, participants were allocated to the basic or the elaborate track of the intervention [38] (for further information, see Supplement 3)."

"The EMIcompass EMI offered tasks based on three different delivery schemes (see [38] for the full manual): (1) Enhancing tasks introduced one new compassion-focused task per week. (2) Consolidating tasks sent user-defined daily reminders to practice tasks that were previously introduced and gave the possibility to complete exercises on-demand. They were added based on meta-analytic evidence indicating that homework compliance contributes to symptom reduction in cognitive behavior therapy studies [42-44]. (3) Interactive tasks were offered to guide participants to use previously learned techniques in moments of high stress or negative affect as detected by voluntary additional EMA. This reflects an important rationale of an EMI: offering tasks in the flow of daily life at a time when the user needs them the most [9, 15]. In each delivery scheme, participants were able to decline the EMI tasks."

5-ix) Describe use parameters

Describe use parameters (e.g., intended "doses" and optimal timing for use). Clarify what instructions or recommendations were given to the user, e.g., regarding timing, frequency, heaviness of use, if any, or was the intervention used ad libitum.

	1	2	3	4	5	
subitem not at all important	○	○	○	○	○	essential

Does your paper address subitem 5-ix?

Copy and paste relevant sections from the manuscript (include quotes in quotation marks "like this" to indicate direct quotes from your manuscript), or elaborate on this item by providing additional information not in the ms, or briefly explain why the item is not applicable/relevant for your study

"The EMIcompass intervention consisted of a 6-week compassion-focused EMI and 4 biweekly sessions (3 training sessions and one review session) as well as an optional on-demand session with a duration of 45-60 minutes."

"EMA data of momentary affect and stress was assessed six times per day, on 3 consecutive days per week during the intervention period."

5-x) Clarify the level of human involvement

Clarify the level of human involvement (care providers or health professionals, also technical assistance) in the e-intervention or as co-intervention (detail number and expertise of professionals involved, if any, as well as “type of assistance offered, the timing and frequency of the support, how it is initiated, and the medium by which the assistance is delivered”. It may be necessary to distinguish between the level of human involvement required for the trial, and the level of human involvement required for a routine application outside of a RCT setting (discuss under item 21 – generalizability).

	1	2	3	4	5	
subitem not at all important	○	○	○	○	○	essential

Does your paper address subitem 5-x?

Copy and paste relevant sections from the manuscript (include quotes in quotation marks "like this" to indicate direct quotes from your manuscript), or elaborate on this item by providing additional information not in the ms, or briefly explain why the item is not applicable/relevant for your study

"The 'EMIcompass' intervention, a transdiagnostic, compassion-focused, hybrid ecological momentary intervention for improving resilience in youth with early mental health problems, was specifically designed building on previous findings on EMA and EMI [4, 14, 32] as well as transdiagnostic momentary mechanisms [13, 24, 33-35] to target psychological distress, clinical symptoms, and candidate mechanisms [36, 37]. It was delivered within a six-week period in addition to treatment as usual to individuals allocated to the experimental condition [36-39] by trained psychologists under regular supervision."

"The EMIcompass intervention consisted of a 6-week compassion-focused EMI and 4 biweekly sessions (3 training sessions and one review session) as well as an optional on-demand session with a duration of 45-60 minutes."

5-xi) Report any prompts/reminders used

Report any prompts/reminders used: Clarify if there were prompts (letters, emails, phone calls, SMS) to use the application, what triggered them, frequency etc. It may be necessary to distinguish between the level of prompts/reminders required for the trial, and the level of prompts/reminders for a routine application outside of a RCT setting (discuss under item 21 – generalizability).

	1	2	3	4	5	
subitem not at all important	○	○	○	○	○	essential

Does your paper address subitem 5-xi? *

Copy and paste relevant sections from the manuscript (include quotes in quotation marks "like this" to indicate direct quotes from your manuscript), or elaborate on this item by providing additional information not in the ms, or briefly explain why the item is not applicable/relevant for your study

"The EMIcompass EMI offered tasks based on three different delivery schemes (see [38] for the full manual): (1) Enhancing tasks introduced one new compassion-focused task per week. (2) Consolidating tasks sent user-defined daily reminders to practice tasks that were previously introduced and gave the possibility to complete exercises on-demand. They were added based on meta-analytic evidence indicating that homework compliance contributes to symptom reduction in cognitive behavior therapy studies [42-44]. (3) Interactive tasks were offered to guide participants to use previously learned techniques in moments of high stress or negative affect as detected by voluntary additional EMA. This reflects an important rationale of an EMI: offering tasks in the flow of daily life at a time when the user needs them the most [9, 15]. In each delivery scheme, participants were able to decline the EMI tasks."

"EMA data of momentary affect and stress was assessed six times per day, on 3 consecutive days per week during the intervention period."

"The threshold for triggering interactive tasks was either high momentary negative affect operationalized as a score of 4 or higher on a 7-point Likert scale ranging from 1-7 or high momentary stress operationalized as a score <0 on a bipolar scale ranging from −3 to 3."

5-xii) Describe any co-interventions (incl. training/support)

Describe any co-interventions (incl. training/support): Clearly state any interventions that are provided in addition to the targeted eHealth intervention, as ehealth intervention may not be designed as stand-alone intervention. This includes training sessions and support [1]. It may be necessary to distinguish between the level of training required for the trial, and the level of training for a routine application outside of a RCT setting (discuss under item 21 – generalizability.

	1	2	3	4	5	
subitem not at all important	○	○	○	○	○	essential

Does your paper address subitem 5-xii? *

Copy and paste relevant sections from the manuscript (include quotes in quotation marks "like this" to indicate direct quotes from your manuscript), or elaborate on this item by providing additional information not in the ms, or briefly explain why the item is not applicable/relevant for your study

The intervention was hybrid and included an EMI plus sessions with a trained psychologist as described above.

6a) Completely defined pre-specified primary and secondary outcome measures, including how and when they were assessed

Does your paper address CONSORT subitem 6a? *

Copy and paste relevant sections from the manuscript (include quotes in quotation marks "like this" to indicate direct quotes from your manuscript), or elaborate on this item by providing additional information not in the ms, or briefly explain why the item is not applicable/relevant for your study

The current study is a secondary analysis.

"EMA data of momentary affect and stress was assessed six times per day, on 3 consecutive days per week during the intervention period."

6a-i) Online questionnaires: describe if they were validated for online use and apply CHERRIES items to describe how the questionnaires were designed/deployed

If outcomes were obtained through online questionnaires, describe if they were validated for online use and apply CHERRIES items to describe how the questionnaires were designed/deployed [9].

	1	2	3	4	5	
subitem not at all important	○	○	○	○	○	essential

Does your paper address subitem 6a-i?

Copy and paste relevant sections from manuscript text

The momentary outocmes for our secondary anaylsis of an exploratory RCT were assessed using EMA. Baseline characteristics were assessed in the baseline appointment. Appointments were held either in the Central Institute of Mental Health or online using video conference tools.

"Self-reported age and gender (i.e., female, male, non-binary) were assessed and clinical staging (i.e., current psychological distress, clinical high at-risk mental state, or first treated episode of severe mental disorder) was rated by blinded assessors based on standardized interviews, self-report, and observer ratings at baseline."

"EMA data of momentary affect and stress was assessed six times per day, on 3 consecutive days per week during the intervention period. "

"Momentary negative affect was assessed using seven established and validated EMA items (Reininghaus et al, 2016): “I feel guilty”, “I feel insecure”, “I feel anxious”, “I feel angry”, “I feel down”, “I doubt myself”, “I am disappointed by myself”. These were rated on a 7-point Likert scale ranging from 1 (=not at all) to 7 (=very much). Momentary stress was operationalized as minor disturbances and distinctive unpleasant events, activities, and social situations in daily life in accordance with previous EMA studies [24]. Participants were asked to report the pleasantness of the most important event that has happened since the last EMA on a bipolar 7-point Likert scale ranging from -3 (= very unpleasant) to +3 (= very pleasant) as well as the pleasantness of the activity they performed just before the EMA and the pleasantness of their current social contact on 7-point Likert scales ranging from 1 (= very unpleasant) to 7 (= very pleasant). Additionally, they were asked to answer the questions “I’m taking care of myself / I’m taking care of somebody” and “I would prefer to be alone / I would prefer to have company” (1=not at all, 7=very much) as follow-up questions to “Who am I with?”. "

"Momentary stress reactivity (i.e., the association between stress and negative affect) was computed in a linear mixed model, with the composite momentary stress measure as the independent variable and negative affect as the outcome variable. From this model, the generated fitted value was fitted for quantifying momentary stress reactivity (Paetzold et al., 2021; Reininghaus, Kempton, et al., 2016). To minimize social desirability bias, all participants were instructed in detail before and during the EMA assessment."

6a-ii) Describe whether and how “use” (including intensity of use/dosage) was defined/measured/monitored

Describe whether and how “use” (including intensity of use/dosage) was defined/measured/monitored (logins, logfile analysis, etc.). Use/adoption metrics are important process outcomes that should be reported in any ehealth trial.

	1	2	3	4	5	
subitem not at all important	○	○	○	○	○	essential

Does your paper address subitem 6a-ii?

Copy and paste relevant sections from manuscript text

For our analyses we defined task-completion, which could be interpreted as active use of an intervention component.

"During the EMI period, passive usage data, including date and time when a task was delivered, the delivery scheme (i.e., enhancing, consolidating, interactive), and the task type (i.e., self-compassionate writing, experiencing emotions as a wave, discovering their own compassionate self, imagining a compassionate companion, counting their breath, breathing with breaks, finding a calming colour, writing a compassionate message, imagining a safe and calm place, and emotional compass) were recorded automatically. Based on this data, we computed the variable “task completion” to indicate an interactive task has been fully completed in the app (i.e., a binary variable, coded 1= yes, 0 = no)."

6a-iii) Describe whether, how, and when qualitative feedback from participants was obtained

Describe whether, how, and when qualitative feedback from participants was obtained (e.g., through emails, feedback forms, interviews, focus groups).

	1	2	3	4	5	
subitem not at all important	○	○	○	○	○	essential

Does your paper address subitem 6a-iii?

Copy and paste relevant sections from manuscript text

"In order to incorporate the participants' experiences with EMIcompass in the research process and to inform the development of a definite trial, a process evaluation was performed [39]."

6b) Any changes to trial outcomes after the trial commenced, with reasons

Does your paper address CONSORT subitem 6b? *

Copy and paste relevant sections from the manuscript (include quotes in quotation marks "like this" to indicate direct quotes from your manuscript), or elaborate on this item by providing additional information not in the ms, or briefly explain why the item is not applicable/relevant for your study

The outcomes used in this paper were defined and preregistered after the exploratory RCT was finished. Deviations from the preregistration are reported in Supplement 1.

7a) How sample size was determined

NPT: When applicable, details of whether and how the clustering by care provides or centers was addressed

7a-i) Describe whether and how expected attrition was taken into account when calculating the sample size

Describe whether and how expected attrition was taken into account when calculating the sample size.

	1	2	3	4	5	
subitem not at all important	○	○	○	○	○	essential

Does your paper address subitem 7a-i?

Copy and paste relevant sections from manuscript title (include quotes in quotation marks "like this" to indicate direct quotes from your manuscript), or elaborate on this item by providing additional information not in the ms, or briefly explain why the item is not applicable/relevant for your study

"For the primary analysis of the EMIcompass RCT, the required sample size was determined with a power simulation [37]. No sample size calculation for our secondary analysis was performed."

7b) When applicable, explanation of any interim analyses and stopping guidelines

Does your paper address CONSORT subitem 7b? *

Copy and paste relevant sections from the manuscript (include quotes in quotation marks "like this" to indicate direct quotes from your manuscript), or elaborate on this item by providing additional information not in the ms, or briefly explain why the item is not applicable/relevant for your study

As this paper is a secondary analysis, this item is not applicable.

8a) Method used to generate the random allocation sequence

NPT: When applicable, how care providers were allocated to each trial group

Does your paper address CONSORT subitem 8a? *

Copy and paste relevant sections from the manuscript (include quotes in quotation marks "like this" to indicate direct quotes from your manuscript), or elaborate on this item by providing additional information not in the ms, or briefly explain why the item is not applicable/relevant for your study

No randomization of participants was performed as described in section Methods; Randomization and Blinding.

"The current secondary analysis used within-subject data generated in the experimental condition of the EMIcompass RCT. Participants in the EMIcompass RCT were randomized at the person-level at a 50:50 ratio to the experimental or control condition. An independent researcher performed block randomization in blocks of four through a computer-generated sequence, with stratification for the three stages (i.e., stages 1a, 1b, and 2). For the current secondary analysis, no further randomization was performed. The criterion whether or not participants completed an EMI task was not randomized but participants decided this voluntarily for each initiated interactive task."

8b) Type of randomisation; details of any restriction (such as blocking and block size)

Does your paper address CONSORT subitem 8b? *

Copy and paste relevant sections from the manuscript (include quotes in quotation marks "like this" to indicate direct quotes from your manuscript), or elaborate on this item by providing additional information not in the ms, or briefly explain why the item is not applicable/relevant for your study

"The current secondary analysis used within-subject data generated in the experimental condition of the EMIcompass RCT. Participants in the EMIcompass RCT were randomized at the person-level at a 50:50 ratio to the experimental or control condition. An independent researcher performed block randomization in blocks of four through a computer-generated sequence, with stratification for the three stages (i.e., stages 1a, 1b, and 2). For the current secondary analysis, no further randomization was performed. The criterion whether or not participants completed an EMI task was not randomized but participants decided this voluntarily for each initiated interactive task."

9) Mechanism used to implement the random allocation sequence (such as sequentially numbered containers), describing any steps taken to conceal the sequence until interventions were assigned

Does your paper address CONSORT subitem 9? *

Copy and paste relevant sections from the manuscript (include quotes in quotation marks "like this" to indicate direct quotes from your manuscript), or elaborate on this item by providing additional information not in the ms, or briefly explain why the item is not applicable/relevant for your study

"The current secondary analysis used within-subject data generated in the experimental condition of the EMIcompass RCT. Participants in the EMIcompass RCT were randomized at the person-level at a 50:50 ratio to the experimental or control condition. An independent researcher performed block randomization in blocks of four through a computer-generated sequence, with stratification for the three stages (i.e., stages 1a, 1b, and 2). For the current secondary analysis, no further randomization was performed. The criterion whether or not participants completed an EMI task was not randomized but participants decided this voluntarily for each initiated interactive task."

10) Who generated the random allocation sequence, who enrolled participants, and who assigned participants to interventions

Does your paper address CONSORT subitem 10? *

Copy and paste relevant sections from the manuscript (include quotes in quotation marks "like this" to indicate direct quotes from your manuscript), or elaborate on this item by providing additional information not in the ms, or briefly explain why the item is not applicable/relevant for your study

"The current secondary analysis used within-subject data generated in the experimental condition of the EMIcompass RCT. Participants in the EMIcompass RCT were randomized at the person-level at a 50:50 ratio to the experimental or control condition. An independent researcher performed block randomization in blocks of four through a computer-generated sequence, with stratification for the three stages (i.e., stages 1a, 1b, and 2). For the current secondary analysis, no further randomization was performed. The criterion whether or not participants completed an EMI task was not randomized but participants decided this voluntarily for each initiated interactive task."

11a) If done, who was blinded after assignment to interventions (for example, participants, care providers, those assessing outcomes) and how

NPT: Whether or not administering co-interventions were blinded to group assignment

11a-i) Specify who was blinded, and who wasn't

Specify who was blinded, and who wasn't. Usually, in web-based trials it is not possible to blind the participants [1, 3] (this should be clearly acknowledged), but it may be possible to blind outcome assessors, those doing data analysis or those administering co-interventions (if any).

	1	2	3	4	5	
subitem not at all important	○	○	○	○	○	essential

Does your paper address subitem 11a-i? *

Copy and paste relevant sections from the manuscript (include quotes in quotation marks "like this" to indicate direct quotes from your manuscript), or elaborate on this item by providing additional information not in the ms, or briefly explain why the item is not applicable/relevant for your study

This is a secondary analysis of an existing exploratory RCT. All relevant information regarding the exploratory RCT for our current analyses are provided in the Methods section. "The current secondary analysis used within-subject data generated in the experimental condition of the EMIcompass RCT. Participants in the EMIcompass RCT were randomized at the person-level at a 50:50 ratio to the experimental or control condition. An independent researcher performed block randomization in blocks of four through a computer-generated sequence, with stratification for the three stages (i.e., stages 1a, 1b, and 2). For the current secondary analysis, no further randomization was performed. The criterion whether or not participants completed an EMI task was not randomized but participants decided this voluntarily for each initiated interactive task."

11a-ii) Discuss e.g., whether participants knew which intervention was the "intervention of interest" and which one was the "comparator"

Informed consent procedures (4a-ii) can create biases and certain expectations - discuss e.g., whether participants knew which intervention was the "intervention of interest" and which one was the "comparator".

	1	2	3	4	5	
subitem not at all important	○	○	○	○	○	essential

Does your paper address subitem 11a-ii?

Copy and paste relevant sections from the manuscript (include quotes in quotation marks "like this" to indicate direct quotes from your manuscript), or elaborate on this item by providing additional information not in the ms, or briefly explain why the item is not applicable/relevant for your study

Participants were informed whether or not they were allocated to the experimental or control condition after randomization. Since we only include data from aprticipants in the experimental condition and no further randomization was performed for this subsample, this item does not apply for our secondary analysis.

11b) If relevant, description of the similarity of interventions

(this item is usually not relevant for ehealth trials as it refers to similarity of a placebo or sham intervention to a active medication/intervention)

Does your paper address CONSORT subitem 11b? *

Copy and paste relevant sections from the manuscript (include quotes in quotation marks "like this" to indicate direct quotes from your manuscript), or elaborate on this item by providing additional information not in the ms, or briefly explain why the item is not applicable/relevant for your study

This item is not applicable as there was no other intervention but the EMIcompass intervention.

12a) Statistical methods used to compare groups for primary and secondary outcomes

NPT: When applicable, details of whether and how the clustering by care providers or centers was addressed

Does your paper address CONSORT subitem 12a? *

Copy and paste relevant sections from the manuscript (include quotes in quotation marks "like this" to indicate direct quotes from your manuscript), or elaborate on this item by providing additional information not in the ms, or briefly explain why the item is not applicable/relevant for your study

The statistical methods for this secondary analysis are described in the sections Methods, Statistical Methods.

12a-i) Imputation techniques to deal with attrition / missing values

Imputation techniques to deal with attrition / missing values: Not all participants will use the intervention/comparator as intended and attrition is typically high in ehealth trials. Specify how participants who did not use the application or dropped out from the trial were treated in the statistical analysis (a complete case analysis is strongly discouraged, and simple imputation techniques such as LOCF may also be problematic [4]).

	1	2	3	4	5	
subitem not at all important	○	○	○	○	○	essential

Does your paper address subitem 12a-i? *

Copy and paste relevant sections from the manuscript (include quotes in quotation marks "like this" to indicate direct quotes from your manuscript), or elaborate on this item by providing additional information not in the ms, or briefly explain why the item is not applicable/relevant for your study

The statistical methods for this secondary analysis are described in the sections Methods, Statistical Methods.

Information on missingness is provided in Supplement 7.

12b) Methods for additional analyses, such as subgroup analyses and adjusted analyses

Does your paper address CONSORT subitem 12b? *

Copy and paste relevant sections from the manuscript (include quotes in quotation marks "like this" to indicate direct quotes from your manuscript), or elaborate on this item by providing additional information not in the ms, or briefly explain why the item is not applicable/relevant for your study

"To examine the extent to which the total number of tasks completed was associated with momentary negative affect, stress, or stress reactivity at the subsequent EMA after interactive tasks completion, we fitted linear mixed models with momentary negative affect, momentary stress, or momentary stress reactivity at the subsequent EMA each entered in separate models as dependent variable and cumulative training frequency as independent variable while controlling for momentary negative affect, stress, or stress reactivity, respectively, at the preceding EMA, time between EMAs, clinical staging, age, gender, context change between EMAs and task time. To examine differences in active use by task type among participants, we performed descriptive analyses by calculating distributions of relative frequencies by task type. For each participant, we calculated the relative frequencies with which they selected the available tasks and then computed mean scores of those frequencies across all participants. We further examined whether, for those who completed interactive tasks in moments of high stress or negative affect, proximal effects on momentary negative affect, momentary stress, and momentary stress reactivity differed depending on task type of interactive tasks. Therefore, overarching task types were summarized by content (i.e., reflection task (emotional compass); breathing exercises (counting their breath, breathing with breaks) and soothing imagery (finding a calming color, imagining a safe and calm place, experiencing emotions as a wave); self-compassion tasks (self-compassionate writing, discovering their own compassionate self, imagining a compassionate companion, writing a compassionate message). We fitted linear mixed models with momentary negative affect, momentary stress, and momentary stress reactivity at the subsequent EMA each entered in separate models as dependent variable and task type (effect-coded and dummy-coded) as independent variable while controlling for momentary outcomes at the preceding EMA, time between EMAs, clinical staging, age, gender, context change between EMAs and task time. See Supplement 3 for more statistical details on exploratory analyses."

X26) REB/IRB Approval and Ethical Considerations [recommended as subheading under "Methods"] (not a CONSORT item)

X26-i) Comment on ethics committee approval

	1	2	3	4	5	
subitem not at all important	○	○	○	○	○	essential

Does your paper address subitem X26-i?

Copy and paste relevant sections from the manuscript (include quotes in quotation marks "like this" to indicate direct quotes from your manuscript), or elaborate on this item by providing additional information not in the ms, or briefly explain why the item is not applicable/relevant for your study

"Ethical Considerations
The EMIcompass RCT received ethical approval from the local ethics committee of the Medical Faculty Mannheim, Heidelberg University (2017-602N-MA). Participants (and for minors participants as well as their caregivers or legal guardians) were asked to provide informed consent. Participation in the study was voluntary and participants were able to withdraw consent at any given time. Data were treated confidentially and analyzed in a pseudonymized form. Data storage and processing were performed in accordance with the General Data Protection Regulation. Participants were covered by liability and commuting accident insurance for the duration of the study. They received compensation for their time (range 85-175€) and travel expenses, depending on how many times they interacted in the EMAs at the outcome assessments (usage of the EMAs or EMI during the intervention period was not compensated). Identification of individual participants is not possible from any part of the manuscript or supplementary material."

x26-ii) Outline informed consent procedures

Outline informed consent procedures e.g., if consent was obtained offline or online (how? Checkbox, etc.?), and what information was provided (see 4a-ii). See [6] for some items to be included in informed consent documents.

	1	2	3	4	5	
subitem not at all important	○	○	○	○	○	essential

Does your paper address subitem X26-ii?

Copy and paste relevant sections from the manuscript (include quotes in quotation marks "like this" to indicate direct quotes from your manuscript), or elaborate on this item by providing additional information not in the ms, or briefly explain why the item is not applicable/relevant for your study

"Participants (and for minors participants as well as their caregivers or legal guardians) were asked to provide informed consent. Participation in the study was voluntary and participants were able to withdraw consent at any given time."

X26-iii) Safety and security procedures

Safety and security procedures, incl. privacy considerations, and any steps taken to reduce the likelihood or detection of harm (e.g., education and training, availability of a hotline)

	1	2	3	4	5	
subitem not at all important	○	○	○	○	○	essential

Does your paper address subitem X26-iii?

Copy and paste relevant sections from the manuscript (include quotes in quotation marks "like this" to indicate direct quotes from your manuscript), or elaborate on this item by providing additional information not in the ms, or briefly explain why the item is not applicable/relevant for your study

"Data were treated confidentially and analyzed in a pseudonymized form. Data storage and processing were performed in accordance with the General Data Protection Regulation. Participants were covered by liability and commuting accident insurance for the duration of the study."

"Identification of individual participants is not possible from any part of the manuscript or supplementary material."

RESULTS

13a) For each group, the numbers of participants who were randomly assigned, received intended treatment, and were analysed for the primary outcome

NPT: The number of care providers or centers performing the intervention in each group and the number of patients treated by each care provider in each center

Does your paper address CONSORT subitem 13a? *

Copy and paste relevant sections from the manuscript (include quotes in quotation marks "like this" to indicate direct quotes from your manuscript), or elaborate on this item by providing additional information not in the ms, or briefly explain why the item is not applicable/relevant for your study

The number and flow of participants is depicted in Figure 1. As this secondary analysis did not compare groups but included only participants in the experimental condition, the item does not fully apply, which is reported in the section Methods; Randomization and Blinding.

13b) For each group, losses and exclusions after randomisation, together with reasons

Does your paper address CONSORT subitem 13b? (NOTE: Preferably, this is shown in a CONSORT flow diagram) *

Copy and paste relevant sections from the manuscript (include quotes in quotation marks "like this" to indicate direct quotes from your manuscript), or elaborate on this item by providing additional information not in the ms, or briefly explain why the item is not applicable/relevant for your study

The number and flow of participants is depicted in Figure 1.

13b-i) Attrition diagram

Strongly recommended: An attrition diagram (e.g., proportion of participants still logging in or using the intervention/comparator in each group plotted over time, similar to a survival curve) or other figures or tables demonstrating usage/dose/engagement.

	1	2	3	4	5	
subitem not at all important	○	○	○	○	○	essential

Does your paper address subitem 13b-i?

Copy and paste relevant sections from the manuscript or cite the figure number if applicable (include quotes in quotation marks "like this" to indicate direct quotes from your manuscript), or elaborate on this item by providing additional information not in the ms, or briefly explain why the item is not applicable/relevant for your study

The intervention is no longer active and was only available to participants during the intervention. Therefore, this item does not apply.

14a) Dates defining the periods of recruitment and follow-up

Does your paper address CONSORT subitem 14a? *

Copy and paste relevant sections from the manuscript (include quotes in quotation marks "like this" to indicate direct quotes from your manuscript), or elaborate on this item by providing additional information not in the ms, or briefly explain why the item is not applicable/relevant for your study

"The EMIcompass RCT was conducted from August 2019 to September 2021 and has been registered in the German Clinical Trials Register (DRKS00017265; https://www.drks.de/drks_web/navigate.do?navigationId=trial.HTML&TRIAL_ID=DRKS00017265) on the 31st July 2019. The first participant was enrolled on 7th August 2019."

14a-i) Indicate if critical “secular events” fell into the study period

Indicate if critical “secular events” fell into the study period, e.g., significant changes in Internet resources available or “changes in computer hardware or Internet delivery resources”

	1	2	3	4	5	
subitem not at all important	○	○	○	○	○	essential

Does your paper address subitem 14a-i?

Copy and paste relevant sections from the manuscript (include quotes in quotation marks "like this" to indicate direct quotes from your manuscript), or elaborate on this item by providing additional information not in the ms, or briefly explain why the item is not applicable/relevant for your study

No critical secular events occurred during study. As we do not believe that the occurence or non-occurance of critical secular events would bias the findings of this secondary analysis, we did not report this in the manuscript.

14b) Why the trial ended or was stopped (early)

Does your paper address CONSORT subitem 14b? *

Copy and paste relevant sections from the manuscript (include quotes in quotation marks "like this" to indicate direct quotes from your manuscript), or elaborate on this item by providing additional information not in the ms, or briefly explain why the item is not applicable/relevant for your study

The exploratory RCT that the data for this secondary analysis is drawn from stopped once the recruitment and data collection was finished.

15) A table showing baseline demographic and clinical characteristics for each group

NPT: When applicable, a description of care providers (case volume, qualification, expertise, etc.) and centers (volume) in each group

Does your paper address CONSORT subitem 15? *

Copy and paste relevant sections from the manuscript (include quotes in quotation marks "like this" to indicate direct quotes from your manuscript), or elaborate on this item by providing additional information not in the ms, or briefly explain why the item is not applicable/relevant for your study

Sample Characteristics are provided in Table 1.

15-i) Report demographics associated with digital divide issues

In ehealth trials it is particularly important to report demographics associated with digital divide issues, such as age, education, gender, social-economic status, computer/Internet/ehealth literacy of the participants, if known.

	1	2	3	4	5	
subitem not at all important	○	○	○	○	○	essential

Does your paper address subitem 15-i? *

Copy and paste relevant sections from the manuscript (include quotes in quotation marks "like this" to indicate direct quotes from your manuscript), or elaborate on this item by providing additional information not in the ms, or briefly explain why the item is not applicable/relevant for your study

Age and gender are reported in Table 1.

16) For each group, number of participants (denominator) included in each analysis and whether the analysis was by original assigned groups

16-i) Report multiple “denominators” and provide definitions

Report multiple “denominators” and provide definitions: Report N’s (and effect sizes) “across a range of study participation [and use] thresholds” [1], e.g., N exposed, N consented, N used more than x times, N used more than y weeks, N participants “used” the intervention/comparator at specific pre-defined time points of interest (in absolute and relative numbers per group). Always clearly define “use” of the intervention.

	1	2	3	4	5	
subitem not at all important	○	○	○	○	○	essential

Does your paper address subitem 16-i? *

Copy and paste relevant sections from the manuscript (include quotes in quotation marks "like this" to indicate direct quotes from your manuscript), or elaborate on this item by providing additional information not in the ms, or briefly explain why the item is not applicable/relevant for your study

There were no groups in this study, thus only the overall number of participants was mentioned.

16-ii) Primary analysis should be intent-to-treat

Primary analysis should be intent-to-treat, secondary analyses could include comparing only "users", with the appropriate caveats that this is no longer a randomized sample (see 18-i).

	1	2	3	4	5	
subitem not at all important	○	○	○	○	○	essential

Does your paper address subitem 16-ii?

Copy and paste relevant sections from the manuscript (include quotes in quotation marks "like this" to indicate direct quotes from your manuscript), or elaborate on this item by providing additional information not in the ms, or briefly explain why the item is not applicable/relevant for your study

This item is not applicable four the current secondary analysis.

17a) For each primary and secondary outcome, results for each group, and the estimated effect size and its precision (such as 95% confidence interval)

Does your paper address CONSORT subitem 17a? *

Copy and paste relevant sections from the manuscript (include quotes in quotation marks "like this" to indicate direct quotes from your manuscript), or elaborate on this item by providing additional information not in the ms, or briefly explain why the item is not applicable/relevant for your study

For any results of the current secondary analysis, we reportet effect sizes as well as 95% confidence intervals.

17a-i) Presentation of process outcomes such as metrics of use and intensity of use

In addition to primary/secondary (clinical) outcomes, the presentation of process outcomes such as metrics of use and intensity of use (dose, exposure) and their operational definitions is critical. This does not only refer to metrics of attrition (13-b) (often a binary variable), but also to more continuous exposure metrics such as “average session length”. These must be accompanied by a technical description how a metric like a “session” is defined (e.g., timeout after idle time) [1] (report under item 6a).

	1	2	3	4	5	
subitem not at all important	○	○	○	○	○	essential

Does your paper address subitem 17a-i?

Copy and paste relevant sections from the manuscript (include quotes in quotation marks "like this" to indicate direct quotes from your manuscript), or elaborate on this item by providing additional information not in the ms, or briefly explain why the item is not applicable/relevant for your study

Provided in Supplement 7.

17b) For binary outcomes, presentation of both absolute and relative effect sizes is recommended

Does your paper address CONSORT subitem 17b? *

Copy and paste relevant sections from the manuscript (include quotes in quotation marks "like this" to indicate direct quotes from your manuscript), or elaborate on this item by providing additional information not in the ms, or briefly explain why the item is not applicable/relevant for your study

No binary outcomes were used in the current analysis.

18) Results of any other analyses performed, including subgroup analyses and adjusted analyses, distinguishing pre-specified from exploratory

Does your paper address CONSORT subitem 18? *

Copy and paste relevant sections from the manuscript (include quotes in quotation marks "like this" to indicate direct quotes from your manuscript), or elaborate on this item by providing additional information not in the ms, or briefly explain why the item is not applicable/relevant for your study

Described in the Results section and named as "exploratory analyses".

18-i) Subgroup analysis of comparing only users

A subgroup analysis of comparing only users is not uncommon in ehealth trials, but if done, it must be stressed that this is a self-selected sample and no longer an unbiased sample from a randomized trial (see 16-iii).

	1	2	3	4	5	
subitem not at all important	○	○	○	○	○	essential

Does your paper address subitem 18-i?

Copy and paste relevant sections from the manuscript (include quotes in quotation marks "like this" to indicate direct quotes from your manuscript), or elaborate on this item by providing additional information not in the ms, or briefly explain why the item is not applicable/relevant for your study

No subgroup analyses performed, but we did conduct exploratory analyses of same-day post-task associations using a broader inclusion criterion (i.e., including all observations for which at least one EMA was completed on the same day after task completion.

"Further, we conducted exploratory analyses of same-day post-task associations using a broader inclusion criterion, including all observations for which at least one EMA was completed on the same day after task completion (see Supplement 2)."

19) All important harms or unintended effects in each group
(for specific guidance see CONSORT for harms)

Does your paper address CONSORT subitem 19? *

Copy and paste relevant sections from the manuscript (include quotes in quotation marks "like this" to indicate direct quotes from your manuscript), or elaborate on this item by providing additional information not in the ms, or briefly explain why the item is not applicable/relevant for your study

"Any adverse events were monitored throughout the study period. Overall, no serious adverse events (i.e., any serious incidents that result in death, persistent or significant disability or incapacity that require hospitalization, or life-threatening situations), and only few adverse device effects (e.g., problems with study smartphone or the app), were observed during the trial period. A more detailed overview can be found elsewhere [36]."

19-i) Include privacy breaches, technical problems

Include privacy breaches, technical problems. This does not only include physical “harm” to participants, but also incidents such as perceived or real privacy breaches [1], technical problems, and other unexpected/unintended incidents. “Unintended effects” also includes unintended positive effects [2].

	1	2	3	4	5	
subitem not at all important	○	○	○	○	○	essential

Does your paper address subitem 19-i?

Copy and paste relevant sections from the manuscript (include quotes in quotation marks "like this" to indicate direct quotes from your manuscript), or elaborate on this item by providing additional information not in the ms, or briefly explain why the item is not applicable/relevant for your study

"Any adverse events were monitored throughout the study period. Overall, no serious adverse events (i.e., any serious incidents that result in death, persistent or significant disability or incapacity that require hospitalization, or life-threatening situations), and only few adverse device effects (e.g., problems with study smartphone or the app), were observed during the trial period. A more detailed overview can be found elsewhere [36]."

19-ii) Include qualitative feedback from participants or observations from staff/researchers

Include qualitative feedback from participants or observations from staff/researchers, if available, on strengths and shortcomings of the application, especially if they point to unintended/unexpected effects or uses. This includes (if available) reasons for why people did or did not use the application as intended by the developers.

	1	2	3	4	5	
subitem not at all important	○	○	○	○	○	essential

Does your paper address subitem 19-ii?

Copy and paste relevant sections from the manuscript (include quotes in quotation marks "like this" to indicate direct quotes from your manuscript), or elaborate on this item by providing additional information not in the ms, or briefly explain why the item is not applicable/relevant for your study

In the current analysis, we report subjective ratings of participants' satisfaction with the different tasks assessed at post-intervention reported in the current study (Supplementary Table S12). No further qualitative feedback was used in the current study. We refer to a different publication analysing qualitative feedback for a process evaluation of the EMIcompass intervention.

"In order to incorporate the participants’ experiences with EMIcompass in the research process and to inform the development of a definite trial, a process evaluation was performed [39]."

DISCUSSION

22) Interpretation consistent with results, balancing benefits and harms, and considering other relevant evidence

NPT: In addition, take into account the choice of the comparator, lack of or partial blinding, and unequal expertise of care providers or centers in each group

22-i) Restate study questions and summarize the answers suggested by the data, starting with primary outcomes and process outcomes (use)

Restate study questions and summarize the answers suggested by the data, starting with primary outcomes and process outcomes (use).

	1	2	3	4	5	
subitem not at all important	○	○	○	○	○	essential

Does your paper address subitem 22-i? *

Copy and paste relevant sections from the manuscript (include quotes in quotation marks "like this" to indicate direct quotes from your manuscript), or elaborate on this item by providing additional information not in the ms, or briefly explain why the item is not applicable/relevant for your study

This item is adressed in the section Discussion; Principal Findings.

22-ii) Highlight unanswered new questions, suggest future research

Highlight unanswered new questions, suggest future research.

	1	2	3	4	5	
subitem not at all important	○	○	○	○	○	essential

Does your paper address subitem 22-ii?

Copy and paste relevant sections from the manuscript (include quotes in quotation marks "like this" to indicate direct quotes from your manuscript), or elaborate on this item by providing additional information not in the ms, or briefly explain why the item is not applicable/relevant for your study

Addressed in the section Discussion; Interpretation and Discussion; Conclusion

"While our current analyses allow for no such conclusion, they underscore the need for future research. Especially in EMA data, even effects in the small effect size range may indicate clinically meaningful change in mental health mechanisms and outcomes, as effects may accumulate over time [75]."

"However, possible differences by symptom severity may be considered in future research."

"Given our limited sample size any potential differences between tasks should be interpreted with caution but rather explored further in future research."

"However, the current analysis does not allow for conclusions to be drawn about the relationship between proximal and distal outcomes. Further evidence on within-person shifts in EMI studies and their potential relevance for longer-term outcomes is needed. Rather than relying solely on between group comparisons to detect whether or not an intervention was effective, modelling individual trajectories may help our understanding of mechanisms in the pathways of change. Larger, more diverse samples and micro-randomized or N-of-1 designs are now required to delineate dose-by-timing interactions and to guide the optimal tailoring, scalable implementation, and equitable use of digital interventions to improve youth mental health."

20) Trial limitations, addressing sources of potential bias, imprecision, and, if relevant, multiplicity of analyses

20-i) Typical limitations in ehealth trials

Typical limitations in ehealth trials: Participants in ehealth trials are rarely blinded. Ehealth trials often look at a multiplicity of outcomes, increasing risk for a Type I error. Discuss biases due to non-use of the intervention/usability issues, biases through informed consent procedures, unexpected events.

	1	2	3	4	5	
subitem not at all important	○	○	○	○	○	essential

Does your paper address subitem 20-i? *

Copy and paste relevant sections from the manuscript (include quotes in quotation marks "like this" to indicate direct quotes from your manuscript), or elaborate on this item by providing additional information not in the ms, or briefly explain why the item is not applicable/relevant for your study

This item is addressed in the section Discussion; Limitations

21) Generalisability (external validity, applicability) of the trial findings

NPT: External validity of the trial findings according to the intervention, comparators, patients, and care providers or centers involved in the trial

21-i) Generalizability to other populations

Generalizability to other populations: In particular, discuss generalizability to a general Internet population, outside of a RCT setting, and general patient population, including applicability of the study results for other organizations

	1	2	3	4	5	
subitem not at all important	○	○	○	○	○	essential

Does your paper address subitem 21-i?

Copy and paste relevant sections from the manuscript (include quotes in quotation marks "like this" to indicate direct quotes from your manuscript), or elaborate on this item by providing additional information not in the ms, or briefly explain why the item is not applicable/relevant for your study

This item is not directly applicable as the aim of the secondary analysis was not to evaluate the intervention. This is discussed in the Discussion section, e.g. "While our analyses with the available data may help our understanding of micro-level effects and processes of CFT-based EMIs such as EMIcompass, this approach is not optimal for evaluating the effectiveness of such interventions."

21-ii) Discuss if there were elements in the RCT that would be different in a routine application setting

Discuss if there were elements in the RCT that would be different in a routine application setting (e.g., prompts/reminders, more human involvement, training sessions or other co-interventions) and what impact the omission of these elements could have on use, adoption, or outcomes if the intervention is applied outside of a RCT setting.

	1	2	3	4	5	
subitem not at all important	○	○	○	○	○	essential

Does your paper address subitem 21-ii?

Copy and paste relevant sections from the manuscript (include quotes in quotation marks "like this" to indicate direct quotes from your manuscript), or elaborate on this item by providing additional information not in the ms, or briefly explain why the item is not applicable/relevant for your study

This item is not applicable as the current study was not an RCT.

OTHER INFORMATION

23) Registration number and name of trial registry

Does your paper address CONSORT subitem 23? *

Copy and paste relevant sections from the manuscript (include quotes in quotation marks "like this" to indicate direct quotes from your manuscript), or elaborate on this item by providing additional information not in the ms, or briefly explain why the item is not applicable/relevant for your study

The current analysis was not a registered RCT, we, however, refer to the registration of the EMIcompass exploratory RCT.

"Trial registration: This study uses data from the EMIcompass exploratory RCT that was prospectively registered in the German Clinical Trials Register (DRKS00017265;https://www.drks.de/drks_web/navigate.do?navigationId=trial.HTML&TRIAL_ID=DRKS00017265)."

"The EMIcompass RCT was conducted from August 2019 to September 2021 and has been registered in the German Clinical Trials Register (DRKS00017265; https://www.drks.de/drks_web/navigate.do?navigationId=trial.HTML&TRIAL_ID=DRKS00017265) on the 31st July 2019."

24) Where the full trial protocol can be accessed, if available

Does your paper address CONSORT subitem 24? *

Cite a Multimedia Appendix, other reference, or copy and paste relevant sections from the manuscript (include quotes in quotation marks "like this" to indicate direct quotes from your manuscript), or elaborate on this item by providing additional information not in the ms, or briefly explain why the item is not applicable/relevant for your study

The current analysis is not a registered RCT, we refer to the protocol paper of the ecploratory RCT. Further, this secondary analysis was preregistered as described in the Methods section.

25) Sources of funding and other support (such as supply of drugs), role of funders

Does your paper address CONSORT subitem 25? *

Copy and paste relevant sections from the manuscript (include quotes in quotation marks "like this" to indicate direct quotes from your manuscript), or elaborate on this item by providing additional information not in the ms, or briefly explain why the item is not applicable/relevant for your study

"This work was supported by the German Research Foundation (DFG) [grant number 389626655] and DFG Heisenberg professorship [grant number 389624707] to UR. The work is further supported by the German Center for Mental Health (DZPG)."

X27) Conflicts of Interest (not a CONSORT item)

X27-i) State the relation of the study team towards the system being evaluated

In addition to the usual declaration of interests (financial or otherwise), also state the relation of the study team towards the system being evaluated, i.e., state if the authors/evaluators are distinct from or identical with the developers/sponsors of the intervention.

	1	2	3	4	5	
subitem not at all important	○	○	○	○	○	essential

Does your paper address subitem X27-i?

Copy and paste relevant sections from the manuscript (include quotes in quotation marks "like this" to indicate direct quotes from your manuscript), or elaborate on this item by providing additional information not in the ms, or briefly explain why the item is not applicable/relevant for your study

"We declare no competing interests."

About the CONSORT EHEALTH checklist

As a result of using this checklist, did you make changes in your manuscript? *

- ○ yes, major changes
- ◉ yes, minor changes
- ○ no

What were the most important changes you made as a result of using this checklist?

The most important changes were made regarding the Abstract and the Methods section.

How much time did you spend on going through the checklist INCLUDING making changes in your manuscript *

The time spent going through this checklist was aproximately 22 hours.

As a result of using this checklist, do you think your manuscript has improved? *

- ◉ yes
- ○ no
- ○ Sonstiges:

Would you like to become involved in the CONSORT EHEALTH group?

This would involve for example becoming involved in participating in a workshop and writing an "Explanation and Elaboration" document

- ○ yes
- ◉ no
- ○ Sonstiges:

Auswahl löschen

Any other comments or questions on CONSORT EHEALTH

Meine Antwort

STOP - Save this form as PDF before you click submit

To generate a record that you filled in this form, we recommend to generate a PDF of this page (on a Mac, simply select "print" and then select "print as PDF") before you submit it.

When you submit your (revised) paper to JMIR, please upload the PDF as supplementary file.

Don't worry if some text in the textboxes is cut off, as we still have the complete information in our database. Thank you!

Final step: Click submit !

Click submit so we have your answers in our database!

Senden

Alle Eingaben löschen

Gib niemals Passwörter über Google Formulare weiter.

Dieser Inhalt wurde nicht von Google erstellt und wird von Google auch nicht unterstützt. - Eigentümer dieses Formulars kontaktieren - Nutzungsbedingungen - Datenschutzerklärung

Sieht dieses Formular verdächtig aus? Bericht

Google Formulare
