# Supplementary material for: Proximal Effects of a Transdiagnostic, Ecological Momentary Intervention for Enhancing Resilience in Help-Seeking Young People (EMIcompass): Findings From a Secondary Analysis of an Exploratory Randomized Controlled Trial
Source: J Med Internet Res. 2026 Sep 3;28:e77150. doi: 10.2196/77150 (PMC13543658; doi:10.2196/77150)
Supplement: Supplementary file 2 [file jmir-v28-e77150-s001.docx]

## Deviations from the Preregistration

Gugel, J., Paetzold, I., Schick, A., Rauschenberg, C., Hirjak, D., Banaschewski, T., Meyer-Lindenberg, A., Boehnke, J. R., Boecking, B., & Reininghaus, U. (2023). Zooming in: Micro-level processes in a compassion-focused ecological momentary intervention. Open Science Framework - Preregistration. <https://doi.org/https://doi.org/10.17605/OSF.IO/MT8CK>

**Deviations:**

- Sensitivity analyses were originally planned with different liberal exclusion criteria, i.e., we planned to exclude cases only when the two next EMAs after the interactive task were not answered. However, our original strict and liberal exclusion criteria included the exact same sample. This is why we performed exploratory analyses of same-day post-task associations with more liberal exclusion criteria, i.e., we only excluded cases when no subsequent EMA at the same day was answered.
- We originally planned to explore the association between EMI task completion and positive affect. These analyses were statistically not possibly due to multicollinearity and were thus not conducted.
- We did not originally include analyses with debriefing data in our preregistration. However, to enrich our analyses on use by task types by including subjective data on participants’ satisfaction with the different EMI tasks, this data was later included.
- Gender was preregistered as a 3-level factor but was used as a 2-level factor in the analyses as not participant in the sample identified as “non-binary”.
- Cohen’s d-type effect sizes were calculated to improve the interpretability and comparability of effects.
- We used person-mean-centered values for all EMA measures.
- In response to reviewer comments in the revision of this manuscript, we were requested to change our original statistical approach (i.e., an analysis using difference scores) and examine the proximal effect of task completion (versus non-completion) on the subsequent EMA scores of each momentary negative affect, stress, and stress reactivity, while controlling for baseline levels of these measures. We were also advised to apply Kenward-Roger adjustments to calculate more conservative margins of error [1]. For full transparency, findings from our preregistered analyses can be found in Further Analyses on Missingness section in Multimedia Appendix 1.

## Exploratory Analyses of Same-Day Post-Task Associations

We conducted exploratory analyses of same-day post-task associations using a broader inclusion criterion, including all observations for which at least one EMA was completed on the same day after task completion. For all exploratory analyses of same-day post-task associations, mixed-effects REML multilevel regression models with random intercepts were fitted. Multilevel models with random intercepts and slopes were tested against all models and rejected for all models.

|  | *β* | *t* | 95% CI | d-type (95% CI) |
| --- | --- | --- | --- | --- |
| Task completion | -0.06 | -1.12 | -0.17 – 0.04 | -0.10 (-0.26 – 0.07) |
| Negative affect at the preceding EMA | 0.52 | 14.85 | 0.45 – 0.58 | 0.82 (0.67 – 0.98) |
| Time between EMAs | 0.02 | 1.00 | -0.02 – 0.06 | 0.03 (-0.03 – 0.10) |
| Clinical staging: stage 1b | 0.29 | 1.69 | -0.06 – 0.63 | 0.46 (-0.08 – 0.99) |
| Clinical staging: stage 2 | 0.72 | 3.09 | 0.24 – 1.20 | 1.15 (0.40 – 1.91) |
| Age | -0.00 | -0.08 | -0.05 – 0.05 | 0.00 (-0.08 – 0.07) |
| Gender | -0.29 | -1.43 | -0.72 – 0.13 | -0.47 (-1.12 – 0.18) |
| Context change between EMAs | -0.01 | -0.12 | -0.10 – 0.09 | -0.01 (-0.16 – 0.14) |
| Task time | -0.03 | -0.73 | -0.10 – 0.05 | -0.04 (-0.16 – 0.07) |

**Table S1.** Exploratory analysis of same-day post-task associations with momentary negative affect in a linear mixed model.

*Note*. Mixed-effects REML multilevel regression with random intercepts. Momentary negative affect at the subsequent EMA was entered as dependent variable and task completion, negative affect at the preceding EMA, time between EMAs, clinical staging, age, gender, context change between EMAs, and task time were entered as independent variables. All measures were z-standardized. A multilevel model with random intercepts and slopes was tested against this model and rejected. CI = 95% confidence interval. N = 620 observations clustered in 37 participants.

**Table S2.** Exploratory analysis of same-day post-task associations with momentary stress in a linear mixed model.

|  | *β* | *t* | 95% CI | d-type (95% CI) |
| --- | --- | --- | --- | --- |
| Task completion | -0.05 | -0.56 | -0.22 – 0.12 | -0.05 (-0.23 – 0.13) |
| Stress at the preceding EMA | 0.15 | 3.31 | 0.06 – 0.24 | 0.16 (0.06 – 0.26) |
| Time between EMAs | 0.01 | 0.31 | -0.06 – 0.08 | 0.01 (-0.06 – 0.09) |
| Clinical staging: stage 1b | 0.20 | 1.05 | -0.19 – 0.58 | 0.21 (-0.18 – 0.61) |
| Clinical staging: stage 2 | 0.17 | 0.71 | -0.33 – 0.67 | 0.19 (-0.33 – 0.70) |
| Age | 0.00 | 0.10 | -0.05 – 0.06 | 0.00 (-0.05 – 0.06) |
| Gender | 0.11 | 0.51 | -0.33 – 0.55 | 0.12 (-0.33 – 0.57) |
| Context change between EMAs | -0.20 | -2.44 | -0.35 – 0.04 | -0.21 (-0.38 – -0.04) |
| Task time | -0.02 | -0.28 | -0.13 – 0.10 | -0.02 (-0.14 – 0.11) |

*Note*. Mixed-effects REML multilevel regression with random intercepts. Momentary stress at the subsequent EMA was entered as dependent variable and task completion, stress at the preceding EMA, time between EMAs, clinical staging, age, gender, context change between EMAs, and task time were entered as independent variables. All measures were z-standardized. A multilevel model with random intercepts and slopes was tested against this model and rejected. CI = 95% confidence interval. N = 617 observations clustered in 36 participants.

**Table S3.** Exploratory analyses of same-day post-task associations with momentary stress reactivity in a linear mixed model.

|  | *β* | *t* | 95% CI | d-type (95% CI) |
| --- | --- | --- | --- | --- |
| Task completion | -0.01 | -0.19 | -0.10 – 0.08 | -0.01 (-0.12 – 0.10) |
| Stress reactivity at the preceding EMA | 0.16 | 3.86 | 0.08 – 0.24 | 0.19 (0.09 – 0.30) |
| Time between EMAs | 0.00 | 0.03 | -0.04 – 0.04 | 0.00 (-0.05 – 0.05) |
| Clinical staging: stage 1b | 0.49 | 1.74 | -0.08 – 1.07 | 0.60 (-0.09 – 1.28) |
| Clinical staging: stage 2 | 1.13 | 2.83 | -0.31 – 1.94 | 1.53 (0.54 – 2.52) |
| Age | -0.03 | -0.84 | -0.11 – 0.05 | -0.04 (-0.14 – 0.05) |
| Gender | -0.64 | -1.78 | -1.37 – 0.10 | -0.77 (-1.63 – 0.09) |
| Context change between EMAs | -0.06 | -1.48 | -0.15 – 0.02 | -0.08 (-0.18 – 0.03) |
| Task time | 0.00 | 0.02 | -0.06 – 0.06 | 0.00 (-0.08 – 0.08) |

*Note*. Linear mixed model with random intercepts. Momentary stress reactivity at the subsequent EMA was entered as dependent variable and task completion, stress reactivity at the preceding EMA, time between EMAs, clinical staging, age, gender, context change between EMAs, and task time were entered as independent variables. All measures were z-standardized. A multilevel model with random intercepts and slopes was tested against this model and rejected. CI = 95% confidence interval. N = 617 observations clustered in 36 participants.

## Further Information on Exploratory Analyses

**Training Effects on the Immediate Impact of Interactive Task Completion on Outcomes:**

The variable “training time” was calculated to control for the number of days the participants have used the intervention at the specific EMA. In line with previous research on the dose-response relationship of treatment duration and mental health outcomes, we wanted to analyse possible effects of cumulative training frequency [2]. Given that the duration of an intervention and the amount of practice are interrelated, we included training time as a control variable with the aim to disaggregate possible effects.

**Differences in Active Use by Task Type:**

Different tasks were offered to our participants at different times. First, there was a basic study track that every participant had access to. Then, participants were allocated to either the basic or the elaborate track of the intervention based on the trained psychologists’ impression and the participants’ experiences in the first 2 weeks of the intervention. While the basic study track focused on creating feelings of safeness and calmness by introducing breathing techniques and soothing imagery, the elaborate track extended breathing exercises and soothing imagery by introducing self-compassionate imagery and writing. The full analytical sample of N=37 participants had access to the tasks in the general study track. From those, a subsample of N=23 participants were allocated to the basic study track and a subsample of N=14 participants were allocated to the elaborate study track. One participant only answered the enhancing tasks introducing tasks from the general study track, hence had no access to any consolidating tasks from the basic or elaborate study track. This is why sample sizes varied for the different tracks.

**Differences in our Proximal Outcomes Predicted by Task Type Clusters:**

Since tasks were introduced to our participants gradually, the sample sizes of completed tasks varied greatly. Task types were aggregated for these analyses based on theoretical considerations about underlying therapeutic mechanisms of change. We generated four overarching task types based on functional equivalence (i.e., breathing exercises, soothing imagery, self-compassion, and reflection task). First, a linear mixed model with random intercepts was fitted using restricted maximum likelihood (REML) using effect-coded overarching task types (for results, see Table S8), followed by a mixed models with random intercepts with dummy-coded task-types. Reflection task was chosen as reference category due to its estimate being close to 0 in the analyses with effect-coding and since this overarching task type comprised general introductory tasks. Multilevel models with random intercepts and slopes were tested against these models and rejected.

**Debriefings:**

In Debriefing questionnaires, participants were asked to rate their subjective satisfaction with the different EMI tasks rated after the intervention period. All tasks were rated with mean values of 4.45 or higher out of 7.

## Crude Models

The following table shows unadjusted proximal effects of EMI interactive task completion on reductions in the momentary outcomes.

**Table S4**. Proximal effects of task completion on momentary negative affect, stress, and stress reactivity values in unadjusted linear mixed models.

|  | *β* | *t* | 95% CI | N | d-type (95% CI) |
| --- | --- | --- | --- | --- | --- |
|  | Outcome: momentary negative affect | | | |  |
| Task completion | 0.05 | 0.89 | -0.06 – 0.16 | 495 | 0.05 (-0.06 – 0.15) |
|  | Outcome: momentary stress | | | |  |
| Task completion | -0.11 | -1.19 | -0.29 – 0.07 | 494 | -0.12 (-0.32 – 0.08) |
|  | Outcome: momentary stress reactivity | | | |  |
| Task completion | -0.03 | -0.53 | -0.13 – 0.08 | 494 | -0.13 (-0.24 – 0.03) |

*Note*. Linear mixed model with random intercepts. Negative affect, difference in stress, and difference in stress reactivity at the subsequent were entered as dependent variable and task completion was entered as independent variables. All measures were z-standardized. A multilevel model with random intercepts and slopes was tested against this model and rejected. CI = 95% confidence interval. The observations were each clustered in 36 participants.

## Supplementary Exploratory Findings

**Training Effects on the Immediate Impact of Interactive Ttask Completion on Outcomes.**

|  | *β* | *t* | 95% CI | d-type (95% CI) |
| --- | --- | --- | --- | --- |
| Cumulative training frequency | 0.08 | 1.42 | -0.03 – 0.19 | 0.14 (-0.05 – 0.33) |
| Negative affect at the preceding EMA | 0.61 | 16.99 | 0.54 – 0.68 | 1.06 (0.88 – 1.25) |
| Time between EMAs | -0.01 | -0.35 | -0.07 – 0.05 | -0.02 (-0.12 – 0.08) |
| Clinical staging: stage 1b | 0.05 | 0.35 | -0.24 – 0.35 | 0.09 (-0.02 – 0.20) |
| Clinical staging: stage 2 | 0.51 | 2.55 | 0.10 – 0.92 | 0.89 (0.10 – 1.68) |
| Age | -0.01 | -0.33 | -0.05 – 0.04 | -0.01 (-0.08 – 0.06) |
| Gender | -0.14 | -0.84 | -0.50 – 0.21 | -0.25 (-0.85 – 0.35) |
| Context change between EMAs | 0.01 | 0.16 | -0.09 – 0.11 | 0.01 (-0.16 – 0.19) |
| Task time | 0.00 | 0.00 | -0.07 – 0.07 | 0.00 (-0.13 – 0.13) |
| Training time (days since start) | -0.00 | -0.83 | -0.01 – 0.00 | 0.00 (-0.02 – 0.01) |

**Table S5.** Proximal effect of cumulative training frequency on momentary negative affect in a linear mixed model.

*Note*. Linear mixed model with random intercepts. Negative affect at the subsequent EMA was entered as dependent variable and cumulative training frequency, negative affect at the preceding EMA, time between EMAs, clinical staging, age, gender, context change between EMAs, and task time were entered as independent variables. All measures were z-standardized. A multilevel model with random intercepts and slopes was tested against this model and rejected. CI = 95% confidence interval. N = 494 observations clustered in 36 participants.

**Table S6.** Proximal effect of cumulative training frequency on momentary stress in a linear mixed model.

|  | *β* | *t* | 95% CI | d-type (95% CI) |
| --- | --- | --- | --- | --- |
| Cumulative training frequency | 0.03 | 0.30 | -0.14 – 0.20 | 0.03 (-0.16 – 0.22) |
| Stress at the preceding EMA | 0.13 | 2.18 | 0.04 – 0.23 | 0.15 (0.04 – 0.26) |
| Time between EMAs | 0.05 | 0.94 | -0.05 – 0.15 | 0.05 (-0.06 – 0.16) |
| Clinical staging: stage 1b | 0.20 | 1.01 | -0.20 – 0.59 | 0.22 (-0.21 – 0.65) |
| Clinical staging: stage 2 | -0.20 | -0.79 | -0.73 – 0.32 | -0.23 (-0.80 – 0.34) |
| Age | 0.02 | 0.61 | -0.04 – 0.07 | 0.02 (-0.04 – 0.07) |
| Gender | 0.28 | 1.26 | -0.18 – 0.74 | 0.31 (-0.17 – 0.80) |
| Context change between EMAs | -0.14 | -1.59 | -0.31 – 0.03 | -0.15 (-0.34 – 0.04) |
| Task time | 0.00 | 0.00 | -0.12 – 0.12 | 0.00 (-0.13 – 0.14) |
| Training time (days since start) | -0.00 | -0.38 | -0.01 – 0.01 | 0.00 (-0.01 – 0.01) |

*Note.* Linear mixed model with random intercepts. Stress at the subsequent EMA was entered as dependent variable and cumulative training frequency, stress at the preceding EMA, time between EMAs, clinical staging, age, gender, context change between EMAs, and task time were entered as independent variables. All measures were z-standardized. A multilevel model with random intercepts and slopes was tested against this model and rejected. CI = 95% confidence interval. N = 493 observations clustered in 36 participants.

|  | *β* | *t* | 95% CI | d-type (95% CI) |
| --- | --- | --- | --- | --- |
| Cumulative training frequency | -0.03 | -0.65 | -0.14 – 0.07 | -0.04 (-0.16 – 0.08) |
| Stress reactivity at the preceding EMA | 0.16 | 3.67 | 0.08 – 0.25 | 0.19 (0.08 – 0.30) |
| Time between EMAs | 0.02 | 0.78 | -0.03 – 0.08 | 0.03 (-0.04 – 0.09) |
| Clinical staging: stage 1b | 0.47 | 1.58 | -0.13 – 1.07 | 0.55 (-0.14 – 1.24) |
| Clinical staging: stage 2 | 0.99 | 2.37 | 0.14 – 1.84 | 1.17 (0.17 – 2.17) |
| Age | -0.03 | -0.83 | -0.12 – 0.05 | -0.04 (-0.14 – 0.06) |
| Gender | -0.55 | -1.49 | -1.32 – 0.21 | -0.65 (-1.53 – 0.22) |
| Context change between EMAs | -0.04 | -0.72 | -0.13 – 0.06 | -0.04 (-0.15 – 0.07) |
| Task time | -0.00 | -0.07 | -0.07 – 0.07 | 0.00 (-0.08 – 0.08) |
| Training time (days since start) | 0.00 | 0.19 | -0.01 – 0.01 | 0.00 (-0.01 – 0.01) |

**Table S7.** Proximal effect of cumulative training frequency on momentary stress reactivity in a linear mixed model.

*Note*. Linear mixed model with random intercepts. Stress reactivity at the subsequent EMA was entered as dependent variable and cumulative training frequency, stress reactivity at the preceding EMA, time between EMAs, clinical staging, age, gender, context change between EMAs, and task time were entered as independent variables. All measures were z-standardized. A multilevel model with random intercepts and slopes was tested against this model and rejected. CI = 95% confidence interval. N = 493 observations clustered in 36 participants.

**Outcomes Predicted by Task Type Clusters**

**Table S8.** Momentary negative affect predicted by overarching task types in a linear mixed model (effect-coded).

|  | *β* | *t* | 95% CI | d-type (95% CI) |
| --- | --- | --- | --- | --- |
| Reflective task | -0.08 | -0.64 | -0.34 – 0.17 | -0.13 (-0.52 – 0.27) |
| Breathing exercises | -0.21 | -1.59 | -0.47 – 0.05 | -0.32 (-0.72 – 0.08) |
| Soothing imagery | -0.16 | -1.20 | -0.41 – 0.10 | -0.24 (-0.64 – 0.15) |
| Self-compassion | 0 |  |  |  |
| Negative affect at the preceding EMA | 0.64 | 12.02 | 0.53 – 0.74 | 0.98 (0.77 – 1.19) |
| Time between EMAs | 0.00 | 0.08 | -0.10 – 0.10 | 0.01 (-0.15 – 0.16) |
| Clinical staging: stage 1b | 0.15 | 0.84 | -0.23 – 0.53 | 0.23 (0.05 – 0.41) |
| Clinical staging: stage 2 | 0.63 | 2.70 | 0.15 – 1.12 | 0.97 (0.15 – 1.79) |
| Age | 0.00 | 0.05 | -0.05 – 0.05 | 0.00 (-0.07 – 0.07) |
| Gender | -0.13 | -0.67 | -0.55 – 0.28 | -0.20 (-0.81 – 0.40) |
| Context change between EMAs | 0.08 | 0.90 | -0.10 – 0.26 | 0.13 (-0.15 – 0.41) |
| Task time | 0.02 | 0.49 | -0.07 – 0.12 | 0.04 (-0.11 – 0.18) |

*Note*. Linear mixed model with random intercepts. Negative affect at the subsequent was entered as dependent variable and effect-coded task type clusters (i.e., breathing exercises, soothing imagery, self-compassion vs. reflection task), negative affect at the preceding EMA time between EMAs, clinical staging, age, gender, context change between EMAs, and task time were entered as independent variables. All measures were z-standardized. Self-compassion was omitted due to low power. A multilevel model with random intercepts and slopes was tested against this model and rejected. CI = 95% confidence interval. N = 233 observations clustered in 27 participants.

**Table S9.** Momentary negative affect predicted by overarching task types in a linear mixed model.

|  | *β* | *t* | 95% CI | d-type (95% CI) |
| --- | --- | --- | --- | --- |
| Breathing exercises | -0.12 | -1.17 | -0.33 – 0.08 | -0.19 (-0.51 – 0.13) |
| Soothing imagery | -0.07 | -0.68 | -0.29 – 0.14 | -0.11 (-0.44 – 0.22) |
| Self-compassion | 0.08 | 0.64 | -0.17 – 0.34 | 0.13 (-0.27 – 0.52) |
| Negative affect at the preceding EMA | 0.64 | 12.02 | 0.53 – 0.74 | 0.98 (0.77 – 1.19) |
| Time between EMAs | 0.00 | 0.08 | -0.10 – 0.10 | 0.01 (-0.15 – 0.16) |
| Clinical staging: stage 1b | 0.15 | 0.84 | -0.23 – 0.53 | 0.23 (-0.31 – 0.77) |
| Clinical staging: stage 2 | 0.63 | 2.70 | 0.15 – 1.12 | 0.97 (0.26 – 1.69) |
| Age | 0.00 | 0.05 | -0.05 – 0.05 | 0.00 (-0.07 – 0.07) |
| Gender | -0.13 | -0.67 | -0.55 – 0.28 | -0.20 (-0.81 – 0.40) |
| Context change between EMAs | 0.08 | 0.90 | -0.10 – 0.26 | 0.13 (-0.15 – 0.40) |
| Task time | 0.02 | 0.49 | -0.07 – 0.23 | 0.04 (-0.11 – 0.18) |

*Note*. Linear mixed model with random intercepts. Negative affect at the subsequent EMA was entered as dependent variable and task types (i.e. breathing exercises, soothing imagery, self-compassion vs. reflection task), negative affect at the preceding EMA, time between EMAs, clinical staging, age, gender, context change between EMAs, and task time were entered as independent variables. All measures were z-standardized. A multilevel model with random intercepts and slopes was tested against this model and rejected. CI = 95% confidence interval. N = 233 observations clustered in 27 participants.

**Table S10.** Momentary stress predicted by overarching task types in a linear mixed model.

|  | *β* | *t* | 95% CI | d-type (95% CI) |
| --- | --- | --- | --- | --- |
| Breathing exercises | 0.16 | 0.97 | -0.16 – 0.47 | 0.17 (-0.17 – 0.50) |
| Soothing imagery | 0.05 | 0.32 | -0.27 – 0.37 | 0.06 (-0.28 – 0.39) |
| Self-compassion | 0.11 | 0.57 | -0.27 – 0.50 | 0.12 (-0.29 – 0.53) |
| Stress at the preceding EMA | 0.08 | 1.11 | -0.06 – 0.22 | 0.08 (-0.06 – 0.23) |
| Time between EMAs | 0.05 | 0.65 | -0.10 – 0.20 | 0.05 (-0.11 – 0.21) |
| Clinical staging: stage 1b | 0.52 | 2.28 | 0.03 – 1.01 | 0.55 (0.08 – 1.03) |
| Clinical staging: stage 2 | -0.02 | -0.06 | -0.62 – 0.58 | -0.02 (-0.62 – 0.58) |
| Age | 0.04 | 1.26 | -0.03 – 0.10 | 0.04 (-0.02 – 0.11) |
| Gender | 0.16 | 0.62 | -0.37 – 0.68 | 0.17 (-0.36 – 0.70) |
| Context change between EMAs | -0.18 | -1.34 | -0.46 – 0.09 | -0.20 (-0.48 – 0.09) |
| Task time | 0.00 | 0.06 | -0.13 – 0.14 | 0.00 (-0.14 – 0.15) |

*Note*. Linear mixed model with random intercepts. Stress at the subsequent EMA was entered as dependent variable and task types (i.e. breathing exercises, soothing imagery, self-compassion vs. reflection task), stress at the preceding EMA, time between EMAs, clinical staging, age, gender, context change between EMAs, and task time were entered as independent variables. All measures were z-standardized. A multilevel model with random intercepts and slopes was tested against this model and rejected. CI = 95% confidence interval. N = 232 observations clustered in 27 participants.
 **Table S11.** Momentary stress reactivity predicted by overarching task types in a linear mixed model.

|  | *β* | *t* | 95% CI | d-type (95% CI) |
| --- | --- | --- | --- | --- |
| Breathing exercises | 0.08 | 0.82 | -0.11 – 0.28 | 0.10 (-0.14 – 0.34) |
| Soothing imagery | 0.03 | 0.27 | -0.18 – 0.23 | 0.03 (-0.22 – 0.29) |
| Self-compassion | 0.10 | 0.79 | -0.15 – 0.34 | 0.12 (-0.18 – 0.43) |
| Stress reactivity at the preceding EMA | 0.19 | 3.07 | 0.07 – 0.32 | 0.24 (0.08 – 0.40) |
| Time between EMAs | 0.03 | 0.59 | -0.07 – 0.12 | 0.03 (-0.08 – 0.15) |
| Clinical staging: stage 1b | 0.49 | 1.64 | -0.13 – 1.12 | 0.61 (-0.13 – 1.35) |
| Clinical staging: stage 2 | 1.10 | 2.81 | 0.29 – 1.91 | 1.36 (0.36 – 2.36) |
| Age | -0.02 | -0.43 | -0.10 – 0.07 | -0.02 (-0.12 – 0.08) |
| Gender | -0.48 | -1.41 | -1.18 – 0.23 | -0.59 (-1.43 – 0.24) |
| Context change between EMAs | -0.07 | -0.83 | -0.25 – 0.10 | -0.09 (-0.30 – 0.12) |
| Task time | 0.02 | 0.49 | -0.07 – 0.11 | 0.03 (-0.08 – 0.14) |

*Note*. Linear mixed model with random intercepts. Stress reactivity at the subsequent EMA was entered as dependent variable and task types (i.e. breathing exercises, soothing imagery, self-compassion vs. reflection task), stress reactivity at the preceding EMA, time between EMAs, clinical staging, age, gender, context change between EMAs, and task time were entered as independent variables. All measures were z-standardized. A multilevel model with random intercepts and slopes was tested against this model and rejected. CI = 95% confidence interval. N = 232 observations clustered in 27 participants.

**Debriefings**

**Table S12.** Mean and standard deviation of satisfaction of the evaluation of the different tasks.

|  | Mean | SD | N |
| --- | --- | --- | --- |
| Compass of Emotions | 5.0 | 1.36 | 44 |
| Counting your Breath | 5.91 | 1.70 | 44 |
| My Soothing Color | 4.45 | 1.78 | 44 |
| My calm, safe, and peaceful place | 6.07 | 1.15 | 44 |
| Breathing with breaks | 5.36 | 1.60 | 14 |
| Emotion as a wave | 4.94 | 1.83 | 18 |
| My toolbox | 5.25 | 1.34 | 16 |
| Compassionate image | 5.60 | 1.26 | 25 |
| Compassionate self | 5.44 | 1.47 | 25 |
| Compassionate message | 6.00 | 1.05 | 19 |

*Note*. In a debriefing questionnaire, all participants that were randomized to the experimental condition were asked to rate their satisfaction with the EMIcompass intervention. This table shows the participants’ evaluations of each specific task, asked as follows: ”How satisfied were you with the individual exercises?” They rated this question from 1 (not at all) to 7 (very much). A sample of N = 46 participants received the debriefing questionnaires, from which 2 did not fill out any question. Sample sizes vary for the different tasks depending on whether or not the participants answered the specific questions. S.D. = standard deviation.

## Findings from the Preregistered Analyses

Thanks to the feedback from the revision we were able to adapt our statistical analyses. To ensure full transparency, findings from our statistical analyses as originally preregistered are shown in the following tables.

**Findings from the Preregistered Primary Analyses**

|  | *β* | *z* | 95% CI | d-type (95% CI) |
| --- | --- | --- | --- | --- |
| Task completion | 0.13 | 1.36 | -0.06 – 0.31 | 0.14 (-0.06 – 0.35) |
| Time between EMAs | -0.00 | -0.07 | -0.10 – 0.10 | 0.00 (-0.12 – 0.11) |
| Clinical staging: stage 1b | 0.15 | 1.09 | -0.12 – 0.42 | 0.17 (-0.13 – 0.47) |
| Clinical staging: stage 2 | -0.06 | -0.32 | -0.46 – 0.33 | -0.07 (-0.51 – 0.36) |
| Age | 0.00 | 0.27 | -0.03 – 0.04 | 0.01 (-0.43 – 0.44) |
| Gender | -0.11 | -0.71 | -0.43 – 0.20 | -0.13 (-0.48 – 0.22) |
| Context change between EMAs | 0.03 | 0.33 | -0.15 – 0.21 | 0.03 (-0.16 – 0.23) |
| Task time | -0.03 | -0.41 | -0.16 – 0.10 | -0.03 (-0.17 – 0.11) |

**Table S13.** Proximal effect of task completion on reductions in momentary negative affect in a linear mixed model.

*Note*. Mixed-effects REML multilevel regression with random intercepts. Difference in negative affect was entered as dependent variable and task completion, time between EMAs, clinical staging, age, gender, context change between EMAs, and task time were entered as independent variables. All measures were z-standardized. A multilevel model with random intercepts and slopes was tested against this model and rejected. CI = 95% confidence interval. N = 493 observations clustered in 36 participants.

|  | *β* | *z* | 95% CI | d-type (95% CI) |
| --- | --- | --- | --- | --- |
| Task completion | 0.19 | 1.44 | -0.07 – 0.44 | 0.14 (-0.05 – .34) |
| Time between EMAs | -0.10 | -1.39 | -0.24 – 0.04 | -0.08 (-0.19 – 0.03) |
| Clinical staging: stage 1b | -0.09 | -0.58 | -0.41 – 0.22 | -0.07 (-0.31 – 0.17) |
| Clinical staging: stage 2 | 0.35 | 1.39 | -0.15 – 0.85 | 0.27 (-0.11 – 0.65) |
| Age | 0.00 | 0.03 | -0.04 – 0.04 | 0.00 (-0.03 – 0.03) |
| Gender | -0.09 | -0.46 | -0.47 – 0.29 | -0.07 (-0.36 – 0.22) |
| Context change between EMAs | 0.14 | 1.14 | -0.10 – 0.39 | 0.11 (-0.18 – 0.40) |
| Task time | -0.01 | -0.12 | -0.19 – 0.17 | -0.01 (-0.15 – 0.13) |

**Table S14.** Proximal effect of task completion on reductions in momentary stress in a linear mixed model*.*

*Note*. Mixed-effects REML multilevel regression with random intercepts. Difference in stress was entered as dependent variable and task completion, time between EMAs, clinical staging, age, gender, context change between EMAs, and task time were entered as independent variables. All measures were z-standardized. A multilevel model with random intercepts and slopes was tested against this model and rejected. CI = 95% confidence interval. N = 492 observations clustered in 36 participants.

|  | *β* | *z* | 95% CI | d-type (95% CI) |
| --- | --- | --- | --- | --- |
| Task completion | 0.15 | 1.15 | -0.11 – 0.42 | 0.12 (-0.08 – 0.32) |
| Time between EMAs | -0.15 | -1.97 | -0.30 – -0.00 | -0.11 (-0.23 – 0.00) |
| Clinical staging: stage 1b | 0.01 | 0.08 | -0.31 – 0.33 | 0.01 (-0.01 – 0.03) |
| Clinical staging: stage 2 | 0.40 | 1.51 | -0.12 – 0.92 | 0.30 (-0.09 – 0.70) |
| Age | 0.02 | 1.02 | -0.01 – 0.06 | 0.02 (-0.02 – 0.05) |
| Gender | -0.11 | -0.54 | -0.50 – 0.29 | -0.08 (-0.38 – 0.22) |
| Context change between EMAs | 0.08 | 0.58 | -0.18 – 0.34 | 0.06 (-0.14 – 0.26) |
| Task time | -0.06 | -0.63 | -0.26 – 0.13 | -0.05 (-0.19 – 0.10) |

**Table S15.** Proximal effect of task completion on reductions in momentary stress reactivity in a linear mixed model.

*Note*. Mixed-effects REML multilevel regression with random intercepts. Difference in stress reactivity was entered as dependent variable and task completion, time between EMAs, clinical staging, age, gender, context change between EMAs, and task time were entered as independent variables. All measures were z-standardized. A multilevel model with random intercepts and slopes was tested against this model and rejected. CI = 95% confidence interval. N = 492 observations clustered in 36 participants.

**Findings from Sensitivity Analyses from our Preregistered Analyses**

**Table S16.** Sensitivity analysis of the proximal effect of task completion on reductions in momentary negative affect in a linear mixed model.

|  | *β* | *z* | 95% CI | d-type (95% CI) |
| --- | --- | --- | --- | --- |
| Task completion | 0.18 | 1.96 | 0.00 – 0.35 | 0.18 (0.00 – 0.36) |
| Time between EMAs | -0.04 | -1.16 | -0.12 – 0.03 | -0.05 (-0.1 – 0.03) |
| Clinical staging: stage 1b | 0.00 | 0.03 | -0.24 – 0.25 | 0.00 (-0.25 – 0.25) |
| Clinical staging: stage 2 | -0.20 | -1.19 | -0.53 – 0.13 | -0.20 (-0.54 – 0.13) |
| Age | 0.01 | 0.36 | -0.03 – 0.04 | 0.01 (-0.03 – 0.04) |
| Gender | -0.04 | -0.26 | -0.31 – 0.24 | -0.04 (-0.32 – 0.24) |
| Context change between EMAs | 0.03 | 0.35 | -0.14 – 0.20 | 0.03 (-0.14 – 0.21) |
| Task time | -0.01 | -0.17 | -0.14 – 0.12 | -0.01 (-0.14 – 0.12) |

*Note*. Mixed-effects REML multilevel regression with random intercepts. Difference in negative affect was entered as dependent variable and task completion, time between EMAs, clinical staging, age, gender, context change between EMAs, and task time were entered as independent variables. All measures were z-standardized. A multilevel model with random intercepts and slopes was tested against this model and rejected. CI = 95% confidence interval. N = 620 observations clustered in 37 participants.

**Table S17.** Sensitivity analysis of the proximal effect of task completion on reductions in momentary stress in a linear mixed model.

|  | *β* | *z* | 95% CI | Cohen’s d (95% CI) |
| --- | --- | --- | --- | --- |
| Task completion | 0.14 | 1.17 | -0.09 – 0.37 | 0.11 (-0.07 – 0.28) |
| Time between EMAs | -0.02 | -0.35 | -0.12 – 0.08 | -0.01 (-0.09 – 0.06) |
| Clinical staging: stage 1b | -0.05 | -0.34 | -0.35 – 0.25 | -0.04 (-0.27 – 0.19) |
| Clinical staging: stage 2 | -0.07 | -0.32 | -0.48 – 0.35 | -0.05 (-0.37 – 0.27) |
| Age | 0.01 | 0.69 | -0.02 – 0.05 | 0.01 (-0.02 – 0.04) |
| Gender | 0.06 | 0.33 | -0.28 – 0.39 | 0.04 (-0.22 – 0.30) |
| Context change between EMAs | 0.15 | 1.31 | -0.08 – 0.38 | 0.12 (-0.14 – 0.38) |
| Task time | 0.01 | 0.07 | -0.16 – 0.18 | 0.00 (-0.13 – 0.14) |

*Note*. Mixed-effects REML multilevel regression with random intercepts. Difference in stress was entered as dependent variable and task completion, time between EMAs, clinical staging, age, gender, context change between EMAs, and task time were entered as independent variables. All measures were z-standardized. A multilevel model with random intercepts and slopes was tested against this model and rejected. CI = 95% confidence interval. N = 617 observations clustered in 36 participants.

**Table S18.** Sensitivity analysis of the proximal effect of task completion on reductions in momentary stress reactivity in a linear mixed model.

|  | *β* | *z* | 95% CI | Cohen’s d (95% CI) |
| --- | --- | --- | --- | --- |
| Task completion | 0.11 | 0.94 | -0.12 – 0.34 | 0.08 (-0.09 – 0.26) |
| Time between EMAs | -0.04 | -0.82 | -0.15 – 0.06 | -0.03 (-0.11 – 0.04) |
| Clinical staging: stage 1b | 0.01 | 0.04 | -0.27 – 0.28 | 0.00 (-0.21 – 0.21) |
| Clinical staging: stage 2 | 0.10 | 0.48 | -0.30 – 0.50 | 0.07 (-0.23 – 0.38) |
| Age | 0.02 | 1.24 | -0.01 – 0.06 | 0.02 (-0.01 – 0.04) |
| Gender | -0.02 | -0.10 | -0.34 – 0.30 | -0.01 (-0.26 – 0.23) |
| Context change between EMAs | 0.08 | 0.66 | -0.15 – 0.31 | 0.06 (-0.12 – 0.24) |
| Task time | -0.03 | -0.38 | -0.21 – 0.14 | -0.03 (-0.16 – 0.11) |

*Note*. Linear mixed model with random intercepts. Difference in stress reactivity was entered as dependent variable and task completion, time between EMAs, clinical staging, age, gender, context change between EMAs, and task time were entered as independent variables. All measures were z-standardized. A multilevel model with random intercepts and slopes was tested against this model and rejected. CI = 95% confidence interval. N = 617 observations clustered in 36 participants.

**Crude Models from our Preregistered Analyses**

|  | *β* | *z* | 95% CI | N | Cohen’s d (95% CI) |
| --- | --- | --- | --- | --- | --- |
|  | Outcome: momentary negative affect | | | |  |
| Task completion | 0.15 | 1.76 | -0.02 – 0.32 | 495 | 0.17 (-0.02 – 0.36) |
|  | Outcome: momentary stress | | | |  |
| Task completion | 0.18 | 1.53 | -0.05 – 0.42 | 494 | 0.14 (-0.04 – 0.33) |
|  | Outcome: momentary stress reactivity | | | |  |
| Task completion | 0.17 | 1.35 | -0.07 – 0.41 | 494 | 0.12 (-0.05 – 0.30) |

**Table S19**. Proximal effects of task completion on reductions in momentary negative affect, stress, and stress reactivity values in unadjusted linear mixed models.

*Note*. Linear mixed model with random intercepts. Difference in negative affect, difference in stress, and difference in stress reactivity were entered as dependent variable and task completion was entered as independent variables. All measures were z-standardized. A multilevel model with random intercepts and slopes was tested against this model and rejected. CI = 95% confidence interval. The observations were each clustered in 36 participants.

**Findings from our Preregistered Analyses of Training Effects on the Immediate Impact of Interactive Task Completion on Momentary Outcomes**

**Table S20.** Proximal effect of cumulative training frequency on reductions in momentary negative affect in a linear mixed model.

|  | *β* | *z* | 95% CI | Cohen’s d (95% CI) |
| --- | --- | --- | --- | --- |
| Cumulative training frequency | -0.04 | -0.52 | -0.19 – 0.11 | -0.04 (-0.21 – 0.12) |
| Time between EMAs | 0.01 | 0.13 | -0.09 – 0.11 | 0.01 (-0.10 – 0.12) |
| Clinical staging: stage 1b | 0.22 | 1.50 | -0.07 – 0.51 | 0.24 (-0.08 – 0.56) |
| Clinical staging: stage 2 | -0.03 | -0.13 | -0.42 – 0.36 | -0.03 (-0.46 – 0.40) |
| Age | 0.01 | 0.34 | -0.03 – 0.04 | 0.01 (.0.03 – 0.05) |
| Gender | -0.13 | -0.84 | -0.45 – 0.18 | -0.15 (-0.50 – 0.20) |
| Context change between EMAs | 0.02 | 0.26 | -0.15 – 0.20 | 0.03 (-0.17 – 0.22) |
| Task time | -0.07 | -1.12 | -0.20 – 0.05 | -0.08 (-0.22 – 0.06) |
| Training time (days since start) | 0.00 | 0.34 | -0.01 – 0.01 | 0.00 (-0.01 – 0.01) |

*Note*. Linear mixed model with random intercepts. Difference in negative affect was entered as dependent variable and cumulative training frequency, time between EMAs, clinical staging, age, gender, context change between EMAs, and task time were entered as independent variables. All measures were z-standardized. A multilevel model with random intercepts and slopes was tested against this model and rejected. CI = 95% confidence interval. N = 494 observations clustered in 36 participants.

|  | *β* | *z* | 95% CI | Cohen’s d (95% CI) |
| --- | --- | --- | --- | --- |
| Cumulative training frequency | -0.12 | -1.26 | -0.30 – 0.06 | -0.09 (-0.24 – 0.05) |
| Time between EMAs | -0.10 | -1.37 | -0.24 – 0.04 | -0.08 (-0.19 – 0.03) |
| Clinical staging: stage 1b | 0.11 | 0.62 | -0.25 – 0.48 | 0.09 (-0.20 – 0.38) |
| Clinical staging: stage 2 | 0.50 | 1.98 | 0.00 – 0.99 | 0.39 (0.00 – 0.78) |
| Age | -0.00 | -0.11 | -0.04 – 0.04 | 0.00 (-0.03 – 0.03) |
| Gender | -0.11 | -0.56 | -0.49 – 0.27 | -0.09 (-0.38 – 0.21) |
| Context change between EMAs | 0.14 | 1.15 | -0.10 – 0.39 | 0.11 (-0.08 – 0.31) |
| Task time | -0.02 | -0.23 | -0.20 – 0.16 | -0.02 (-0.16 – 0.12) |
| Training time (days since start) | 0.01 | 1.74 | -0.00 – 0.02 | 0.01 (0.00 – 0.02) |

**Table S21.** Proximal effect of cumulative training frequency on reductions in momentary stress in a linear mixed model.

*Note.* Linear mixed model with random intercepts. Difference in stress was entered as dependent variable and cumulative training frequency, time between EMAs, clinical staging, age, gender, context change between EMAs, and task time were entered as independent variables. All measures were z-standardized. A multilevel model with random intercepts and slopes was tested against this model and rejected. CI = 95% confidence interval. N = 493 observations clustered in 36 participants.

**Table S22.** Proximal effect of cumulative training frequency on reductions in momentary stress reactivity in a linear mixed model.

|  | *β* | *z* | 95% CI | Cohen’s d (95% CI) |
| --- | --- | --- | --- | --- |
| Cumulative training frequency | -0.08 | -0.85 | -0.28 – 0.11 | -0.06 (-0.21 – 0.08) |
| Time between EMAs | -0.15 | -1.94 | -0.30 – 0.00 | -0.11 (-0.22 – 0.00) |
| Clinical staging: stage 1b | 0.18 | 0.90 | -0.21 – 0.56 | 0.13 (-0.15 – 0.42) |
| Clinical staging: stage 2 | 0.51 | 1.94 | -0.01 – 1.04 | 0.38 (0.00 – 0.77) |
| Age | 0.02 | 0.94 | -0.02 – 0.06 | 0.02 (-0.02 – 0.05) |
| Gender | -0.13 | -0.64 | -0.53 – 0.27 | -0.10 (-0.13 – -0.06) |
| Context change between EMAs | 0.08 | 0.60 | -0.18 – 0.34 | 0.06 (-0.13 – 0.25) |
| Task time | -0.07 | -0.75 | -0.26 – 0.12 | -0.05 (-0.19 – 0.09) |
| Training time (days since start) | 0.01 | 1.28 | -0.00 – 0.02 | 0.01 (0.00 – 0.02) |

*Note*. Linear mixed model with random intercepts. Difference in momentary stress reactivity was entered as dependent variable and cumulative training frequency, time between EMAs, clinical staging, age, gender, context change between EMAs, and task time were entered as independent variables. All measures were z-standardized. A multilevel model with random intercepts and slopes was tested against this model and rejected. CI = 95% confidence interval. N = 493 observations clustered in 36 participants.

**Findings from our Preregistered Analyses on Outcomes Predicted by Task Type Clusters**

|  | *β* | *z* | 95% CI | d-type (95% CI) |
| --- | --- | --- | --- | --- |
| Breathing exercises | 0.39 | 2.12 | 0.03 – 0.75 | 0.36 (0.02 – 0.70) |
| Soothing imagery | 0.24 | 1.27 | -0.13 – 0.61 | 0.22 (-0.12 – 0.56) |
| Self-compassion | 0.01 | 0.03 | -0.43 – 0.45 | 0.01 (-0.40 – 0.41) |
| Time between EMAs | 0.04 | 0.45 | -0.13 – 0.21 | 0.04 (-0.12 – 0.19) |
| Clinical staging: stage 1b | 0.03 | 0.13 | -0.36 – 0.41 | 0.02 (-0.33 – 0.38) |
| Clinical staging: stage 2 | -0.34 | -1.18 | -0.90 – 0.22 | -0.31 (-0.84 – 0.21) |
| Age | 0.01 | 0.70 | -0.05 – 0.07 | 0.01 (-0.04 – 0.07) |
| Gender | -0.08 | -0.32 | -0.58 – 0.42 | -0.08 (-0.54 – 0.39) |
| Context change between EMAs | -0.13 | -0.85 | -0.44 – 0.17 | -0.12 (-0.40 – 0.16) |
| Task time | -0.11 | -1.32 | -0.26 – 0.05 | -0.10 (-0.24 – 0.05) |

**Table S23.** Reductions in momentary negative affect predicted by overarching task types in a linear mixed model.

*Note*. Linear mixed model with random intercepts. Difference in negative affect was entered as dependent variable and task types (i.e. breathing exercises, soothing imagery, self-compassion vs. reflection task), time between EMAs, clinical staging, age, gender, context change between EMAs, and task time were entered as independent variables. All measures were z-standardized. A multilevel model with random intercepts and slopes was tested against this model and rejected. CI = 95% confidence interval. N = 233 observations clustered in 27 participants.

**Table S24.** Reductions in momentary negative affect predicted by overarching task types in a linear mixed model (effect-coded).

|  | *β* | *z* | 95% CI | Cohen’s d (95% CI) |
| --- | --- | --- | --- | --- |
| Reflective task | -0.01 | -0.03 | -0.45 – 0.43 | -0.01 (-0.41 – 0.40) |
| Breathing exercises | 0.38 | 1.69 | -0.06 – 0.83 | 0.35 (-0.06 – 0.77) |
| Soothing imagery | 0.23 | 1.01 | -0.22 – 0.68 | 0.21 (-0.30 – 0.63) |
| Self-compassion | 0 |  |  |  |
| Time between EMAs | 0.04 | 0.45 | -0.13 – 0.21 | 0.04 (-0.12 – 0.19) |
| Clinical staging: stage 1b | 0.03 | 0.13 | -0.36 – 0.41 | 0.02 (-0.33 – 0.38) |
| Clinical staging: stage 2 | -0.34 | -1.18 | -0.90 – 0.22 | -0.31 (-0.84 – 0.21) |
| Age | 0.01 | 0.39 | -0.05 – 0.07 | 0.01 (-0.04 – 0.07) |
| Gender | -0.08 | -0.32 | -0.58 – 0.42 | -0.08 (-0.54 – 0.39) |
| Context change between EMAs | -0.13 | -0.85 | -0.44 – 0.17 | -0.12 (-0.40 – 0.16) |
| Task time | -0.11 | -1.32 | -0.26 – 0.05 | -0.10 (-0.24 – 0.05) |

*Note*. Linear mixed model with random intercepts. Difference in negative affect was entered as dependent variable and effect-coded task type clusters (i.e., breathing exercises, soothing imagery, self-compassion vs. reflection task), time between EMAs, clinical staging, age, gender, context change between EMAs, and task time were entered as independent variables. All measures were z-standardized. Self-compassion was omitted due to low power. A multilevel model with random intercepts and slopes was tested against this model and rejected. CI = 95% confidence interval. N = 233 observations clustered in 27 participants.

**Table S25.** Reductions in momentary stress predicted by overarching task types in a linear mixed model.

|  | *β* | *z* | 95% CI | Cohen’s d (95% CI) |
| --- | --- | --- | --- | --- |
| Breathing exercises | -0.11 | -0.45 | -0.61 – 0.38 | -0.08 (-0.42 – 0.26) |
| Soothing imagery | 0.16 | 0.62 | -0.34 – 0.65 | 0.11 (-0.24 – 0.45) |
| Self-compassion | -0.21 | -0.70 | -0.81 – 0.38 | -0.15 (-0.56 – 0.27) |
| Time between EMAs | -0.02 | -0.18 | -0.25 – 0.21 | -0.01 (-0.17 – 0.14) |
| Clinical staging: stage 1b | -0.32 | -1.45 | -0.75 – 0.11 | -0.22 (-0.52 – 0.08) |
| Clinical staging: stage 2 | 0.32 | 0.89 | -0.39 – 1.03 | 0.22 (-0.27 – 0.71) |
| Age | -0.01 | -0.24 | -0.09 – 0.07 | -0.01 (-0.06 – 0.05) |
| Gender | -0.17 | -0.53 | -0.81 – 0.46 | -0.12 (-0.56 – 0.32) |
| Context change between EMAs | 0.10 | 0.46 | -0.31 – 0.51 | 0.07 (-0.22 – 0.35) |
| Task time | -0.01 | -0.13 | -0.22 – 0.20 | -0.01 (-0.15 – 0.13) |

*Note*. Linear mixed model with random intercepts. Difference in stress was entered as dependent variable and task types (i.e. breathing exercises, soothing imagery, self-compassion vs. reflection task), time between EMAs, clinical staging, age, gender, context change between EMAs, and task time were entered as independent variables. All measures were z-standardized. A multilevel model with random intercepts and slopes was tested against this model and rejected. CI = 95% confidence interval. N = 232 observations clustered in 27 participants.

**Table S26.** Reductions in momentary stress reactivity predicted by overarching task types in a linear mixed model.

|  | *β* | *z* | 95% CI | Cohen’s d (95% CI) |
| --- | --- | --- | --- | --- |
| Breathing exercises | -0.01 | -0.04 | -0.58 – 0.55 | -0.01 (-0.34 – 0.33) |
| Soothing imagery | 0.21 | 0.73 | -0.36 – 0.78 | 0.13 (-0.21 – 0.47) |
| Self-compassion | -0.24 | -0.68 | -0.92 – 0.45 | -0.14 (-0.55 – 0.27) |
| Time between EMAs | -0.09 | -0.65 | -0.35 – 0.18 | -0.05 (-0.21 – 0.10) |
| Clinical staging: stage 1b | -0.19 | -0.75 | -0.68 – 0.30 | -0.11 (-0.41 – 0.18) |
| Clinical staging: stage 2 | 0.34 | 0.82 | -0.47 – 1.16 | 0.20 (-0.28 – 0.69) |
| Age | 0.02 | 0.43 | -0.07 – 0.11 | 0.01 (-0.04 – 0.06) |
| Gender | -0.18 | -0.49 | -0.91 – 0.54 | -0.11 (-0.54 – 0.32) |
| Context change between EMAs | 0.03 | 0.12 | -0.44 – 0.50 | 0.02 (-0.26 – 0.30) |
| Task time | -0.07 | -0.58 | -0.31 – 0.17 | -0.04 (-0.19 – 0.10) |

*Note*. Linear mixed model with random intercepts. Difference in stress reactivity was entered as dependent variable and task types (i.e. breathing exercises, soothing imagery, self-compassion vs. reflection task), time between EMAs, clinical staging, age, gender, context change between EMAs, and task time were entered as independent variables. All measures were z-standardized. A multilevel model with random intercepts and slopes was tested against this model and rejected. CI = 95% confidence interval. N = 232 observations clustered in 27 participants.

## Further Analyses on Missingness

Participants completed, overall, 1,337 of 3,606 EMAs during the EMI, corresponding to an EMA completion rate of 37.1%. Thus, 2,269 EMAs were not completed, corresponding to an EMA non-completion rate of 62.9%. In addition, 763 interactive EMI tasks were triggered during the intervention period. Of these, 353 tasks were completed and 410 were not completed, corresponding to a task-completion rate of 46.3%.

We performed Little’s MCAR test including all variables relevant to the analyses. The test was significant, χ²(99) = 1531.1050, *p* < .01, indicating that the data were not missing completely at random. To further examine whether missingness in the EMA outcomes was associated with sociodemographic, clinical, or EMA-level variables, we conducted additional multilevel logistic regression analyses. The results of these findings are reported in Table S26, S27, and S28. For missingness in negative affect at the subsequent EMA, the overall model was not significant (χ²(5) = 5.46, p = .36), and none of the included predictors was significantly associated with missingness. For missingness in stress at the subsequent EMA, the overall model was significant (χ²(7) = 25.49, p < .01). In this model, task completion was significantly associated with missingness (*OR* = 0.00, 95% CI = 0.00 – 0.03, *p* < .01), indicating that participants who completed the EMI task were less likely to have missing stress data at the subsequent EMA. For missingness in stress reactivity at the subsequent EMA, the overall model fit was also significant (χ²(7) = 25.00, p < .01). Task completion (*OR* = 0.00, 95% CI = 0.00 – 0.03, *p* < .01) and age (*OR* = 0.83, 95% CI = 0.69 – 0.99, *p* = .04) were significantly associated with missingness.

**Table S27.** Multilevel logistic analyses of missings in momentary negative affect

|  | *OR* | 95% CI | *z* | *p* |
| --- | --- | --- | --- | --- |
| Negative affect at the preceding EMA | 0.80 | 0.51 – 1.24 | -1.01 | 0.31 |
| Task completion | 1 (omitted) | | | |
| Clinical staging: stage 1b | 2.02 | 0.52 – 7.90 | 1.01 | 0.31 |
| Clinical staging: stage 2 | 3.27 | 0.54 – 19.77 | 1.29 | 0.20 |
| Age | 0.87 | 0.73 – 1.04 | -1.50 | 0.14 |
| Gender | 1.03 | 0.25 – 4.24 | 0.04 | 0.97 |
| Context change between EMAs | 1 (omitted) | | | |
| Task time | 1 (omitted) | | | |

*Note*. Missings in negative affect at the subsequent EMA was entered as dependent variable and negative affect at the preceding EMA, clinical staging, age, gender, context change between EMAs, and task time were entered as independent variables. All measures were z-standardized. OR = Odds Ratio. CI = 95% confidence interval. N = 312 observations clustered in 36 participants.

**Table S28.** Multilevel logistic analyses of missings in momentary stress

|  | *OR* | 95% CI | *Z* | *p* |
| --- | --- | --- | --- | --- |
| Negative affect at the preceding EMA | 0.85 | 0.59 – 1.21 | -0.91 | 0.36 |
| Task completion | 0.00 | 0.00 – 0.03 | -4.58 | 0.00 |
| Clinical staging: stage 1b | 2.30 | 0.60 – 8.82 | 1.22 | 0.22 |
| Clinical staging: stage 2 | 2.66 | 0.45 – 15.83 | 1.07 | 0.28 |
| Age | 0.84 | 0.70 – 1.00 | -1.95 | 0.05 |
| Gender | 1.07 | 0.26 – 4.40 | 0.09 | 0.93 |
| Context change between EMAs | 1 (omitted) | | | |
| Task time | 2.21 | 0.48 – 10.05 | 1.02 | 0.31 |

*Note*. Missings in stress at the subsequent EMA was entered as dependent variable and stress at the preceding EMA, clinical staging, age, gender, context change between EMAs, and task time were entered as independent variables. All measures were z-standardized. OR = Odds Ratio. CI = 95% confidence interval. N = 368 observations clustered in 39 participants.

|  | *OR* | 95% CI | *z* | *p* |
| --- | --- | --- | --- | --- |
| Negative affect at the preceding EMA | 0.82 | 0.49 – 1.38 | -0.73 | 0.46 |
| Task completion | 0.00 | 0.00 – 0.03 | -4.56 | 0.00 |
| Clinical staging: stage 1b | 2.44 | 0.61 – 9.82 | 1.26 | 0.21 |
| Clinical staging: stage 2 | 3.21 | 0.51 –20.43 | 1.24 | 0.22 |
| Age | 0.83 | 0.69 – 0.99 | -2.03 | 0.04 |
| Gender | 0.92 | 0.22 – 3.90 | -0.12 | 0.91 |
| Context change between EMAs | 1 (omitted) | | | |
| Task time | 2.23 | 0.49 – 10.26 | 1.03 | 0.30 |

**Table S29.** Multilevel logistic analyses of missings in momentary stress reactivity

*Note*. Missings in stress reactivity at the subsequent EMA was entered as dependent variable and stress reactivity at the preceding EMA, clinical staging, age, gender, context change between EMAs, and task time were entered as independent variables. All measures were z-standardized. OR = Odds Ratio. CI = 95% confidence interval. N = 468 observations clustered in 39 participants.

## Additional References

1.	Kenward MG, Roger JH. Small sample inference for fixed effects from restricted maximum likelihood. Biometrics. 1997:983-97. doi: 10.2307/2533558.

2.	Nordmo M, T. MJ, Andreas HP, and Solbakken OA. Investigating the dose–response effect in open-ended psychotherapy. Psychotherapy Research. 2021 2021/10/03;31(7):859-69. doi: 10.1080/10503307.2020.1861359.
